# Analysis of Metabolite Profiling in Human Endothelial Cells after Plasma Jet Treatment

**DOI:** 10.1155/2019/3015150

**Published:** 2019-11-03

**Authors:** Yanjie Yang, Dehui Xu, Ning Ning, Yujing Xu

**Affiliations:** ^1^Department of Cardiovascular Medicine, First Affiliated Hospital of the Medical School, Xi'an Jiaotong University, Xi'an, Shaanxi 710049, China; ^2^State Key Laboratory of Electrical Insulation and Power Equipment, Centre for Plasma Biomedicine, Xi'an Jiaotong University, Xi'an, Shaanxi 710049, China; ^3^The School of Life Science and Technology, Xi'an Jiaotong University, Xi'an, Shaanxi 710049, China

## Abstract

Cold atmospheric plasma (CAP) is a novel technology, which has been widely applied in biomedicine, especially in wound healing, dermatological treatment, hemostasis, and cancer treatment. In most cases, CAP treatment will interact with innumerable blood capillaries. Therefore, it is important and necessary to understand the effects of CAP treatment on endothelial cell metabolism. In this study, the metabolite profiling of plasma treatment on endothelial cells was measured by gas chromatography tandem time-of-flight mass spectrometry (GC-TOF-MS). We found that 695 signals (metabolites) were detected by GC-TOF-MS and then evaluated using orthogonal projections to latent structures discriminant analysis (OPLS-DA). All the differential metabolites were listed, and proline and xanthosine were the two of the most downregulated metabolites by plasma treatment. By comprehensive metabolic pathway analysis with the KEGG pathway, we showed that alanine, aspartate, glutamate, and purine metabolism pathways were the most significantly suppressed after gas plasma treatment in human endothelial cells. Our finding gives an overall picture of the metabolic pathways affected by plasma treatment in endothelial cells.

## 1. Introduction

Cold atmospheric plasma (CAP) technology breaks through the limitation of the vacuum chamber required by the low-pressure plasma and realizes that the temperature of the plasma is close to the room temperature level, so that it can directly interact on the living body and the human body, thus creating an emerging interdisciplinary subject-plasma medicine [1, 2]. The cold plasma can be generated under atmospheric pressure by high-voltage discharging of gas, which contains various components such as ultraviolet photons, charged particles, metastable particles, and ground-state neutral particles [3, 4]. At present, cold plasma is mainly used in biomedical applications such as sterilization, wound healing, hemostasis, dermatological treatment, and cancer treatment [5–11], and at least three products have passed the US Food and Drug Administration (FDA) certification [12], so it has a very broad application prospect in biomedicine.

Wound healing is the first and only application for accredited plasma devices in clinical field so far [13, 14]. In the process of wound healing by plasma treatment, although the plasma will directly treat the ulcer or the wound area, the vessels and endothelial cells are not directly exposed by plasma, and the generation of various reactive species such as H_2_O_2_ and NO_2_^−^ will permeate and affect the physiology function of endothelial cells. So it is necessary to study the interaction of cold plasma with numerous blood capillaries surrounding in the wound area. Suzuki and Yoshino reported an enhanced proliferation activity of endothelial cells induced by a micropower plasma [15]. They further pointed out that it was an integrated effect of several ROS produced by cold plasma, rather than single H_2_O_2_, which could not induce the proliferation at the same concentration [15]. Plasma might react with enzymes and have some chemical modifications of amino acids and thus alter the cell construction and cell function [16, 17]. In our study, we investigated the interaction of cold plasma with endothelial cells in the aspect of cell metabolism. Cell metabolism is a general term for a series of ordered chemical reactions that occur in living organisms to sustain life. These reaction processes allow organisms to grow and reproduce, maintain their structure, and react to the external environment [18]. On bioinformatic analyzing metabolic profiling in endothelial cells after plasma jet treatment, we could first report the systemic metabolic network in plasma-treated endothelial cells. Based on our experiments and metabolomic analysis, we found that alanine, aspartate, glutamate, and purine metabolism pathways were the most significantly suppressed after gas plasma treatment in human endothelial cells. These findings also indicate that plasma treatment might benefit to those diseases that have an abnormal high level of glutamate and purine metabolism.

## 2. Methods and Materials

### 2.1. Plasma Jet Generation

In this study, we used a gas plasma jet that was used in our previous studies [19, 20]. The plasma jet consists of a 1 mm powered electrode enclosed in a quartz tube, with a grounded outer electrode wrapped around a 6.0 mm diameter dielectric tube. The working gas was He gas with 99.9999% purity. With 10 kHz and *V*pp = 8 kV of a sinusoidal power supply, the He plasma jet could be generated in the atmospheric air with a temperature lower than 30°C.

### 2.2. Optical Emission Spectroscopy

Emission spectroscopy experiments were performed using a spectrometer (Ocean Optics, USB2000) and calibrated with a Model 63356 Oriel NISL light source. The wavelength range of the spectrometer is 300 nm–800 nm, the resolution is 0.015 nm, and the aperture size of the fiber is 500 *μ*m. The optical probe was mounted directly 3 cm away from the plasma jet.

### 2.3. Cell Culture Condition

The HMEC-1 human endothelial cells were purchased from Shanghai Guandao biological engineering company. HMEC-1 cells were cultured in the Roswell Park Memorial Institute (RPMI) 1640 medium with 10% fetal calf serum, 100 U/mL penicillin, and 50 *μ*g/mL streptomycin (Gibco-Invitrogen, CA). Cell were placed at 37°C in an incubator (Thermo Scientific, USA) containing 5% CO_2_. The cells were refreshed in a new medium 24 h before plasma treatment.

### 2.4. Solvents and Reagents

Pyridine and chloroform were purchased from Admas (Shanghai, China). Bistrifluoroacetamide (BSTFA) and 1% trimethyl chloro silane (TMCS, v/v) were from REGIS Technologies Inc (Morton Grove, IL, USA) and methanol (HPLC grade) from CNW Technologies (Shanghai, China). *L*-2-Chlorophenylalanine was from Hengbai Biotechnology Co Ltd (Shanghai, China). Methoxyamine hydrochloride was from TCI company (Shanghai, China). Deionized water (Thermo; Waltham, MA, USA) was used throughout the experiment.

### 2.5. Sample Collection

5 × 10^5^ cells/well were cultured in the 200 *μ*L medium in 24-well plates. Cells were treated with plasma jet (1 cm above the bottom of the well plates) for 20 s in the plasma treatment group, and for control group, the cells were exposed with He gas flow without plasma discharging. After incubation for 24 hours, 24 wells of cells were collected together into one sample and counted to ensure that the number of each sample was about 1 × 10^7^ cells for further mass spectrometer analysis (*n* = 4). Cells were centrifuged at 4°C for 5 min at the speed of 135 g and washed 3 times at 4°C with PBS at the speed of 76 g. Cell mass in the EP tube was placed in liquid nitrogen for 5 min rapidly and stored in −80°C for further analysis.

### 2.6. Measurement of Long-Lived Species by Plasma Treatment

The concentrations of H_2_O_2_, NO_2_^−^, and NO_3_^−^ after plasma jet treatment in the medium without cells were measured using the hydrogen peroxide/peroxidase assay kit (Thermo Fisher Scientific) and nitrite/nitrate colorimetric assay kit (Cayman), respectively. The plasma jet setting up was the same as before, 200 *μ*L medium in 24-well plates was treated with plasma jet for different durations, and species was measured using a microplate reader (Thermo Fisher Scientific).

### 2.7. Metabolite Extraction

All samples were transferred into a 2 mL tube, and 1000 *μ*L precold extraction mixture (methanol/chloroform (v : v) = 3 : 1) with 10 *μ*L internal standard (*L*-2-chlorophenylalanine, 1 mg/mL stock) was added. Samples were vortexed for 30 s and homogenized with ball mill for 4 min at 45 Hz, followed by ultrasonication for 5 min in ice water. After centrifugation at 4°C for 15 min at 12000 rpm, the supernatant was transferred to a fresh tube. After evaporation in a vacuum concentrator, 20 *μ*L of methoxyamination hydrochloride (20 mg/mL in pyridine) was added and then incubated at 80°C for 30 min and then derivatized by 30 *μ*L of the BSTFA regent (1% TMCS, v/v) at 70°C for 1.5 h. All samples were then analyzed by gas chromatography coupled with a time-of-flight mass spectrometer (GC-TOF-MS).

### 2.8. GC-TOF-MS Analysis

Gas chromatography coupled with a time-of-flight mass spectrometer (GC-TOF-MS) analysis was performed using an Agilent 7890 gas chromatograph system. The system utilized a DB-5MS capillary column. 1 *μ*L aliquot of sample was injected in the splitless mode. Helium was used as the carrier gas, the front inlet purge flow was 3 mL·min^−1^, and the gas flow rate through the column was 1 mL·min^−1^. The initial temperature was kept at 50°C for 1 min, then raised to 310°C at a rate of 10°C·min^−1^, and then kept for 8 min at 310°C. The injection, transfer line, and ion source temperatures were 280, 280, and 250°C, respectively. The energy was −70 eV in the electron impact mode. The mass spectrometry data were acquired in the full-scan mode with the *m/z* range of 50–500 at a rate of 12.5 spectra per second after a solvent delay of 6.33 min.

### 2.9. Data Preprocessing, Annotation, and Statistical Analysis

Raw data analysis, including peak extraction, baseline adjustment, deconvolution, alignment, and integration, was finished with Chroma TOF (V 4.3x, LECO) software and LECO-Fiehn Rtx5 database was used for metabolite identification by matching the mass spectrum and retention index. For the GC-TOF-MS analysis, 4 independent experiments were done and samples were detected and analyzed by mass spectrum. For other measurements, experiments were repeated three times and data were presented as means ± SD. Differences between groups were evaluated using Student's *t*-test. *P* < 0.05 was considered statistically significant.

## 3. Results

### 3.1. Plasma Jet and the Emission Spectrum

**Figure 1:**
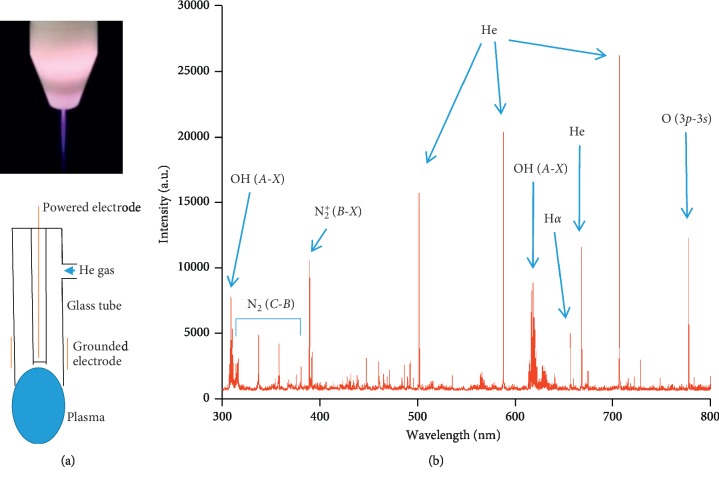
Characters of He plasma jet and the emission profiles: (a) discharge photograph and schematic diagram of the plasma jet; (b) emission spectra of the plasma jet and some of the characteristic spectrum lines.

A sinusoidal power supply with 10 kHz and *V*pp = 8 kV was used to generate our plasma jet at a He gas flow of 2 SLM.  Figure 1(a) shows the discharging photograph of the He plasma jet and the structural profile of the plasma jet. The discharging characters of the He plasma jet were depicted by an emission spectrum, as shown in Figure 1(b). There were several spectral lines in the He plasma jet that were marked in the emission profiles.

### 3.2. Long-Lived Species Produced by Plasma Jet

**Figure 2:**
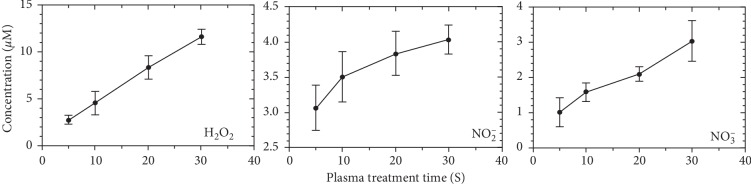
Long-lived species produced by plasma jet. Concentration of H_2_O_2_, NO_2_^−^, and NO_3_^−^ was measured after plasma jet treatment for different durations.

Plasma could generate various reactive species and interact with cells, thus regulating cell metabolism and cell function. Here, we measured several long-lived species in the medium after plasma jet treatment for different durations (Figure 2). In this case, 20 s of plasma jet treatment is a low dose with the concentration of H_2_O_2_ lower than 4 10 *μ*M, which has no cytotoxicity on the HMEC-1 cells (data not shown).

### 3.3. Metabolic Profiling by GC-TOF-MS

**Figure 3:**
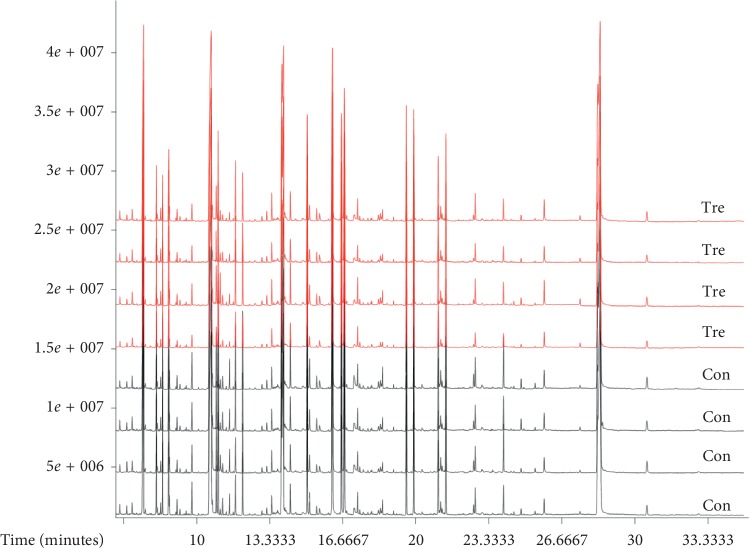
GC-TOF-MS chromatograms by mass spectra. Con represents the control group of cells with He gas only, and Tre group represents cells treated with plasma for 20 s.

A total of 8 samples were investigated. 4 were HMEC-1 cells with gas flow as the control group, and another 4 samples were HMEC-1 cells treated with He plasma for 20 s. With GC-TOF-MS analysis, around 763 signals were detected per sample using mass spectral deconvolution software for peak detection. By normalization with internal standard, finally 695 valid peaks were remained for further analysis.  Figure 3 shows the overall representative GC-TOF-MS chromatograms of the control group and plasma treatment group. The database mapping of metabolites was listed in the additional [Supplementary-material supplementary-material-1].

### 3.4. Orthogonal Projections to Latent Structures Discriminant Analysis (OPLS-DA)

By OPLS-DA analysis, we can filter out the metabolites that are not related to the categorical variables. Through analyzing nonorthogonal variables and orthogonal variables separately, we can obtain more reliable differences about metabolites between the control and experimental groups.

The logarithmic conversion and UV formatting of the data were performed using SIMCA software (V15.0.2, Sartorius Stedim Data Analytics AB, Umea, Sweden). First, the first principal component was subjected to OPLS-DA modeling analysis. The test was performed by 7-fold cross validation; then, the validity of the model was judged by RY (the interpretability of the model for categorical variable *Y*) and *Q* (predictability of the model) obtained by cross-validation; finally, through the permutation test, the order of the categorical variables *Y* is randomly changed several times to obtain different random *Q* values, and the validity of the model is further tested. The cumulative interpretation rate of each group compared to the OPLS-DA model is listed in Table 1.

**Figure 4:**
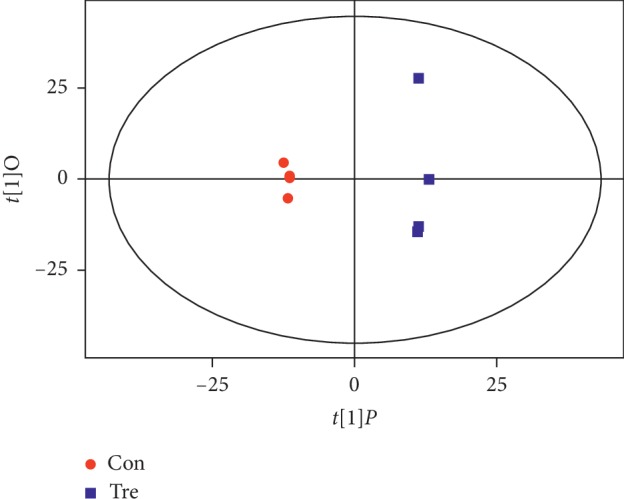
The score scatter plot of the OPLS-DA model.

As Figure 4 shows, the abscissa *t*[1]*P* represents the predicted principal component score of the first principal component, the ordinate *t*[1]O represents the orthogonal principal component score, and the scatter shape and color represent different experimental groups. From the results of the OPLS-DA score chart, it can be seen that the two groups of samples are very significant, and the samples are all in the 95% confidence interval (Hotelling's T-squared ellipse).

**Figure 5:**
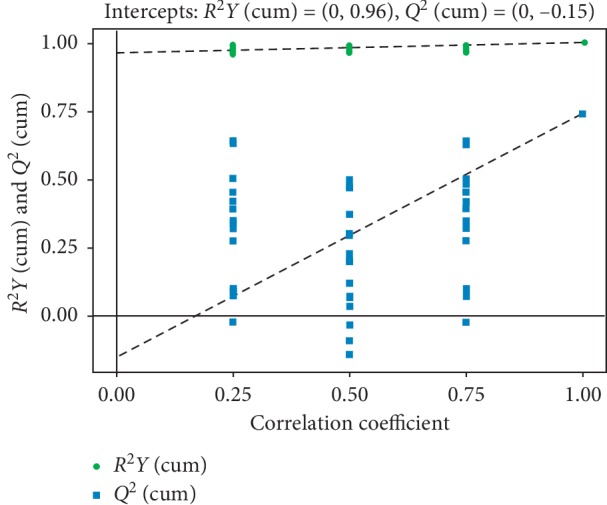
The permutation test of the OPLS-DA model.

The permutation test establishes the corresponding OPLS-DA model by randomly changing the order of the categorical variables *Y* (multiple times *n* = 200) to obtain the *RY* and *Q* values of the stochastic model, avoiding the overfitting of the test model and the statistics of the evaluation model. In general, the original model can better explain the difference between the two sets of samples. The *Q* value of the stochastic model of the displacement test is smaller than the *Q* value of the original model; the intercept of the regression line and the vertical axis of *Q* is less than zero; and as the retention decreases, the proportion of the substituted *Y* variable increases, and the *Q* of the stochastic model gradually decreases (Figure 5). It shows that the original model has good robustness and there is no overfitting phenomenon.

### 3.5. Screening of Differential metabolites

The intrinsic properties of metabolome data based on GC-TOF-MS require us to analyze the data using a multivariate statistical analysis method. Compared with the traditional univariate analysis (UVA) such as Student's *t*-test, analysis of variance (ANOVA), and other independent changes in metabolite levels, statistical analysis of multivariate variables pays more attention to metabolites' relationships and their promotion/antagonism in biological processes. The card value standard used in this project is that Student's *t-*test (*P* value) is less than 0.05, and the variable import importance of the first principal component of the OPLS-DA model (variable importance in the projection (VIP)) greater than 1. The results of differential metabolite screening were described in the control group as an example of the treatment group. The differential metabolite screening results are shown in the additional file 2. Among them, proline and xanthosine were the two of the most downregulated metabolites by plasma treatment.

**Figure 6:**
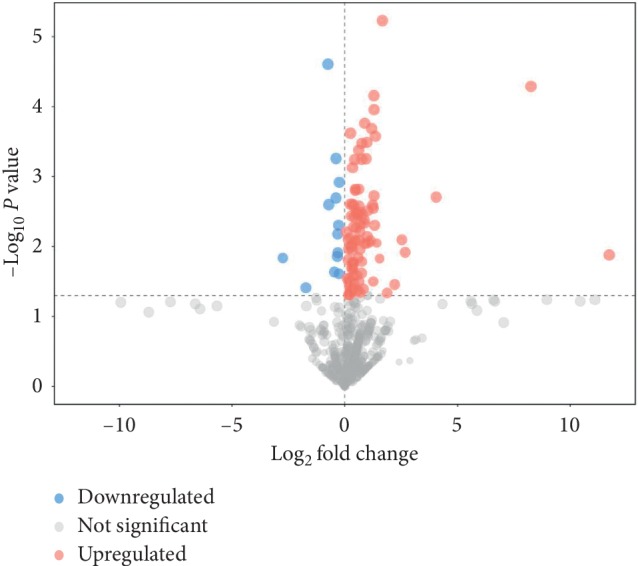
Volcano plot for differential metabolites between control and plasma treatment group. Red represents upregulated metabolites, blue represents downregulated metabolites, and gray represents metabolites that have no significant change.

We visualized the results of screening for differential metabolites in the form of volcano plots (Figure 6). Each point in the volcano map represents a metabolite, the abscissa represents the fold change of the group compared to the substance (take the base 2 logarithm), and the ordinate represents the *P* value of Student's *t*-test (take the negative base 10 logarithm). The scatter size represents the VIP value of the OPLS-DA model. The larger the scatter, the larger the VIP value. The scatter color represents the final screening result, the significantly upregulated metabolites are shown in red, the significantly downregulated metabolites are shown in blue, and the nonsignificantly different metabolites are gray.

### 3.6. Cluster Analysis

**Figure 7:**
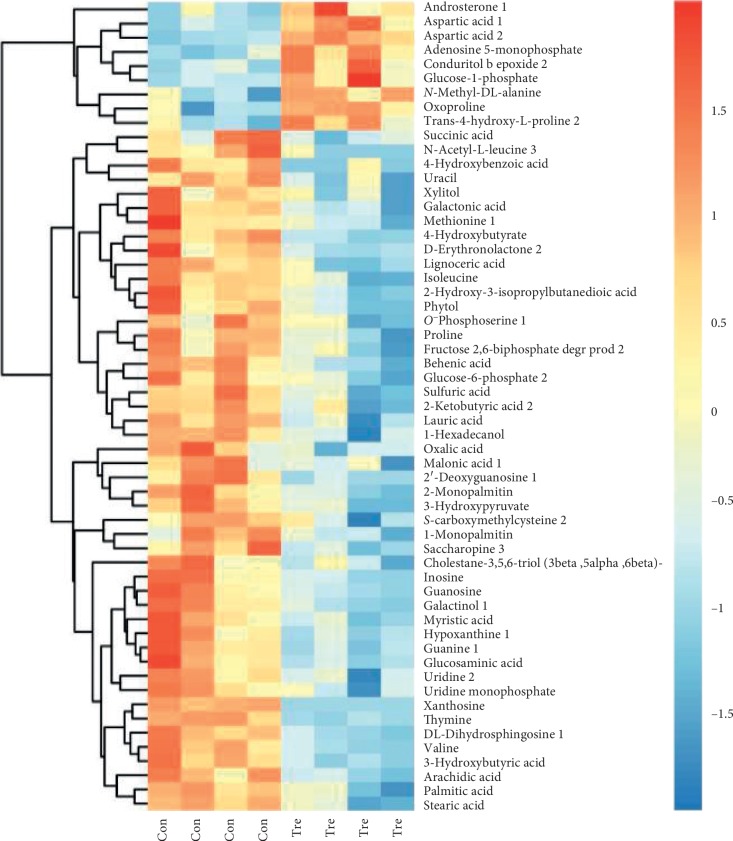
Heatmap of hierarchical clustering analysis for the control and plasma treatment group. Upregulated expressed metabolites are shown in red; downregulated expressed metabolites are shown in blue. ^*∗*^*P* < 0.05.

The differential metabolites obtained by the above analysis often have biologically similar results and functional similarities/complementarities or are positively/negatively regulated by the same metabolic pathway, showing similar or opposite expression characteristics among different experimental groups. Hierarchical clustering analysis of such features helps us classify metabolites with the same characteristics into one class and find the characteristics of metabolites between experimental groups. For each set of comparisons, we calculated the Euclidean distance matrix for the quantitative values of the differential metabolites, clustered the differential metabolites in a completely linked manner, and displayed them in a thermogram (Figure 7). It indicated that there were significant differences in the expression of metabolites between the two groups.

### 3.7. KEGG Analysis of Differential Metabolites

Kyoto Encyclopedia of Genes and Genomes (KEGG) Pathway database (http://www.kegg.jp/kegg/pathway.html) based on functional information of genes and genomes, using metabolic reactions as a clue, concatenation possible metabolic pathways, and corresponding regulatory proteins display the physiological and biochemical processes of the cells in a graphical manner [21]. These processes include energy metabolism, material transport, signal transmission, cell cycle regulation, etc., as well as information on conserved subpathways, and are the most commonly used accessory databases for metabolic network research. We sorted out all pathways for the corresponding metabolite mapping of the Homo sapiens (human), as shown in the additional file 3. After obtaining the above results, we labeled the differential metabolites on the whole KEGG pathway map (Sup [Supplementary-material supplementary-material-1]), with bright red for upregulation and bright blue for downregulation.

### 3.8. Metabolic Pathway Analysis

**Figure 8:**
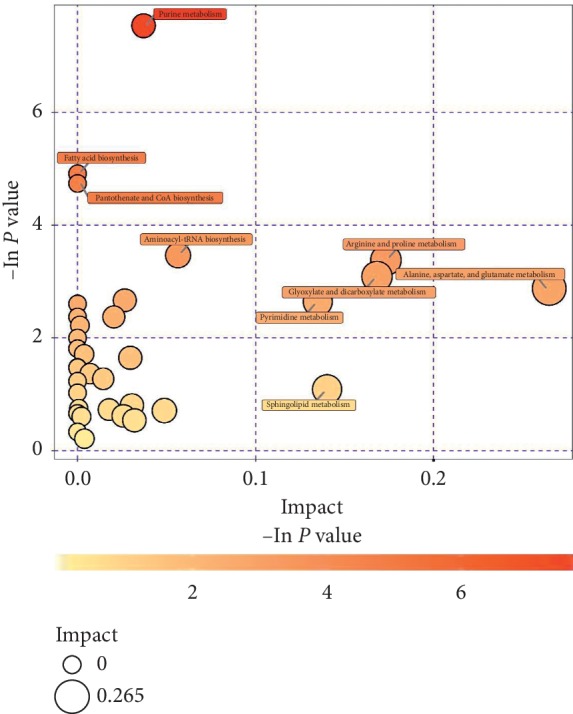
Bubble chart of the metabolic pathway analysis. One bubble represented one metabolic pathway; differentially expressed metabolic pathways have been labeled.

The KEGG annotation analysis only finds pathways in which all differential metabolites are involved, but to understand whether these pathways are closely related to experimental conditions, further metabolic pathway analysis of differential metabolites is required. Through a comprehensive analysis of the pathways of differential metabolites, including enrichment analysis and topological analysis, we can further screen the pathways to find the key pathways that are most relevant to metabolite differences. We first mapped the authoritative metabolite databases such as KEGG and PubChem by differential metabolites. Examples of metabolite mapping tables are shown in the additional file 4. The results of the metabolic pathway analysis are shown in a bubble chart (Figure 8). Each bubble in the bubble diagram represents a metabolic pathway. The abscissa and bubble size of the bubble indicate the size of the influence factor of the pathway in the topological analysis. The larger the size, the larger the influence factor. The ordinate of the bubble and the bubble color indicate the enrichment analysis. The deeper the color, the smaller the *P* value, the more significant the enrichment. From the bubble diagram, we could find out that alanine, aspartate, and glutamate metabolism pathway was the most significantly suppressed after gas plasma treatment in HMEC-1 human endothelial cells. Furthermore, it was worth noting that purine metabolism was also significantly inhibited in endothelial cells.

## 4. Discussion

As a new developed technology, cold atmospheric plasma has aroused widespread concern in biomedical applications. More and more studies revealed the details of the interaction between plasma and cells [22–24]. Plasma produced various short-lived and long-lived species which will affect the biological molecular and regulate cell function. H_2_O_2_ is one of the important long-lived species produced by plasma, but plasma treatment is a comprehensive and synergetic effect of various species, which is quite different from treatment with H_2_O_2_ solution alone. Compared to plasma treatment, corresponding concentration of H_2_O_2_ solution would not have the same effects as plasma [15, 19]. Meanwhile, it is reported that H_2_O_2_ and RNS such as NO_2_^−^ and NO_2_ in the plasma might have chemical reaction and enhance the effects induced by plasma treatment [25–27].

Cell metabolism is a basal process that could indicate the molecular interaction in cells for cell function. Therefore, it is a new perspective to explore changes in metabolites and metabolic pathways before and after plasma treatment. In our study, we investigated the changes in cell metabolism after CAP treatment of endothelial cells by GC-TOF-MS analysis. From results, we found that plasma jet treatment could alter the metabolism profiling in endothelial cells. By multivariate analysis, we screened the differential metabolites that were significantly changed after plasma treatment. Among them, proline and xanthosine were the two of the most downregulated metabolites by plasma treatment. Proline could be oxidized by plasma treatment into three oxidation products corresponding to (Pro-2H + O)H+ at *m/z* 130.05, (Pro + O)H+ at *m/z* 132.07, and (Pro + 2O)H+ at *m/z* 148.06 by mass spectrum as reported by Takai et al. [17]. Xanthosine is a nucleoside derived from xanthine and ribose. Although there is no report about xanthosine could be directly affected by plasma, there were several studies reported that plasma could induce oxidative stress and damage DNA [28, 29]. However, individual metabolite analysis might give us some information about which metabolite is more susceptible to plasma treatment, and the entire metabolism network is complicated and synergistic. For example, some metabolites have similar or opposite functions, so it is better to consider the alteration by comprehensive analysis of certain metabolic pathways. By analyzing the metabolic pathways with KEGG analysis that have the highest correlation with differential metabolites, we reported that alanine, aspartate, and glutamate metabolism had significant change after plasma jet treatment. In the glucose-alanine cycle pathway, glutamate dehydrogenase in muscle catalyzes the binding of *α*-ketoglutaric acid to ammonia to form glutamate, followed by glutamate catalyzed by alanine aminotransferase; pyruvic acid forms alpha-ketoglutarate and alanine [30]. In the bioactive substance metabolism pathway, glutamate itself is an excitatory neurotransmitter, which is widely present in the brain and spinal cord. *γ*-Aminobutyric acid formed by decarboxylation of glutamate is an inhibitory neurotransmitter in organisms widely present in the body [31, 32]. However, in the amino acid synthesis pathway, glutamic acid is an important precursor for the synthesis of glutamine, proline, arginine, and lysine [33, 34]. Cold plasma treatment could suppress glutamate metabolism, which might benefit for diseases that have a high level of glutamate metabolism [35, 36].

In addition, plasma jet treatment can also significantly reduce the purine metabolic pathway in endothelial cells. Purine is a substance in the human body that could cause gout when its metabolism is disordered [37]. After a series of metabolic changes, the resulting product of purine is called uric acid. Uric acid does not have any physiological function in the human body. Under normal circumstances, 2/3 of uric acid produced in the body is excreted by the kidneys and 1/3 is excreted by the large intestine. The body's uric acid is constantly being produced and excreted, so it maintains a certain concentration in the blood [38]. When the concentration of blood uric acid is too high, uric acid is deposited in the joints, soft tissues, cartilage, and kidneys in the form of sodium salts and causes joint swelling, deformity, stiffness, ecchymosis around the joints, nodules, and gouty kidney stones [38, 39]. In a more serious situation, the patients will have gouty renal failure, gouty coronary heart disease, hyperlipidemia, hypertension, urinary calculi, etc. Based on our results, plasma treatment could inhibit the purine metabolism and thus lowers the level of uric acid, which might be benefit for gout-related diseases.

For wound healing that is already applied in clinical, the local effects might dominate the outcomes in plasma treatment. Few seconds of direct plasma treatment could efficiently inactivate the bacteria on the wound, while it would not damage the blood vessels in the tissue. For chronic wound, many times of plasma treatment are necessary, and thus, long-term low-dose plasma treatment could be a potential strategy. In this case, long-term treatment with low concentration of several long-lived species by plasma might be suppressed and would be benefit to those diseases that have abnormal high level of glutamate and purine metabolism. Even more, we might inject the plasma-treated saline solution into the vessels to regulate the abnormal glutamate and purine metabolism. Certainly, we need more experiments and results to further investigate the effects and safety in the animal model.

In conclusion, we analyzed the differential metabolites in endothelial cells after plasma jet treatment by bioinformatics analysis. Furthermore, we found two differential metabolic pathways, glutamate metabolism and purine metabolism pathway, which was vulnerable to plasma treatment, and was significantly suppressed by plasma treatment. Our results indicated that low-dose long-term plasma treatment might benefit those diseases that have abnormal high level of glutamate and purine metabolism.

## Figures and Tables

**Table 1:** Statistical model parameters table of the OPLS-DA model.

Model	Type	*A*	*N*	*R*2*X* (cum)	*R*2*Y* (cum)	*Q*2 (cum)

Model 2	OPLS-DA	1 + 1+ 0	8	0.502	0.997	0.741

## Data Availability

The data sets generated and/or analyzed during the current study are available from the corresponding author on reasonable request.

## Supplementary Materials

Supplementary Materials Additional file 1. Table 1: metabolite mapping. Additional file 2. Table 2: differential metabolites. Additional file 3. Table 3: KEGG pathway. Additional file 4. Table 4: pathway analysis. Sup Figure 1: metabolic pathways with red/blue dots representing the deferentially expressed compounds. Bright red dots represent upregulated metabolites; bright blue dots represent downregulated metabolites.
