# Supplementary material for: Analysis of Metabolite Profiling in Human Endothelial Cells after Plasma Jet Treatment
Source: Biomed Res Int. 2019 Nov 3;2019:3015150. doi: 10.1155/2019/3015150 (PMC6875299; doi:10.1155/2019/3015150)
Supplement: Supplementary Materials — Additional file 1. Table 1: metabolite mapping. Additional file 2. Table 2: differential metabolites. Additional file 3. Table 3: KEGG pathway. Additional file 4. Table 4: pathway analysis. Sup Figure 1: metabolic pathways with red/blue dots representing the deferentially expressed compounds. Bright red dots represent upregulated metabolites; bright blue dots represent downregulated metabolites. [file 3015150.f1.pdf]

Additional file 1

Peak	Analyte	Similarity	R.T. (minutes)	Count	Mass
		NA	Average		
Analyte 1	1	0	6.35267,0	8	57
Analyte 2	2	0	6.36433,0	8	89
Analyte 3	3	0	6.37133,0	7	63
Analyte 4	4	0	6.39033,0	8	57
Analyte 5	5	0	6.43067,0	4	204
Analyte 6	6	0	6.44833,0	4	220
Analyte 7	7	0	6.46089,0	6	86
Analyte 8	8	0	6.463,0	8	281
Analyte 9	9	0	6.49683,0	8	221
Analyte 10	10	0	6.508,0	6	57
Analyte 11	11	0	6.51583,0	8	102
Analyte 12	12	0	6.53917,0	8	147
Analyte 13	13	0	6.54767,0	4	152
Analyte 14	14	0	6.58657,0	7	160
Analyte 15	15	0	6.6524,0	5	143
Analyte 16	16	0	6.66433,0	8	93
Analyte 17	17	0	6.60829,0	7	170
Analyte 18	18	0	6.71133,0	3	93
Analyte 19	19	0	6.71633,0	8	117
Analyte 20	20	0	6.72644,0	3	207
Analyte 21	21	0	6.74267,0	8	57
Analyte 22	22	0	6.74867,0	8	121
Analyte 23	23	0	6.7775,0	8	144
Analyte 24	24	0	6.79133,0	7	191
Analyte 25	25	0	6.78767,0	8	281
Analyte 26	26	0	6.81733,0	8	107
Analyte 27	27	0	6.83233,0	8	57
Analyte 28	28	0	6.8396,0	5	144
Analyte 29	29	0	6.84783,0	8	248
Analyte 30	30	0	6.89133,0	7	207
Analyte 31	31	0	6.92217,0	8	70
Analyte 32	32	0	6.9315,0	8	160
Analyte 33	33	0	6.97817,0	8	71
Analyte 34	34	0	6.99583,0	8	85
Analyte 35	35	0	7.034,0	8	93
Analyte 36	36	0	7.03952,0	7	248
Analyte 37	37	0	7.06333,0	8	147
Analyte 38	38	244	7.06981,0	7	89
Analyte 39	39	0	7.11967,0	8	70

Analyte 40	40	262	7.12333,0	3	172
2-hydroxypyridine	41	282	7.13756,0	6	136
Analyte 42	42	0	7.14383,0	8	102
Analyte 43	43	0	7.1705,0	8	207
Analyte 44	44	225	7.223,0	8	58
Analyte 45	45	281.5	7.25111,0	6	119
Analyte 46	46	0	7.2705,0	8	152
Analyte 47	47	0	7.27467,0	4	318
Analyte 48	48	0	7.30783,0	8	68
Analyte 49	49	0	7.31817,0	8	229
2-ketobutyric acid 2	50	315.375	7.40333,0	8	174
unknown	51	204	7.40767,0	4	56
lactic acid	52	336.66667	7.422,0	8	207
unknown	53	376	7.45667,0	6	158
Analyte 54	54	0	7.48283,0	8	127
Analyte 55	55	0	7.52,0	8	174
unknown	56	541.75	7.52467,0	8	79
glycolic acid	57	573.5	7.57767,0	8	117
unknown	58	161.42857	7.58767,0	8	79
unknown	59	568	7.6265,0	8	190
unknown	60	409.83333	7.63083,0	8	221
Analyte 61	61	0	7.66133,0	4	145
Analyte 62	62	0	7.65733,0	8	71
Analyte 63	63	0	7.66133,0	6	92
Analyte 64	64	0	7.69324,0	7	225
Analyte 65	65	0	7.71183,0	8	89
Analyte 66	66	0	7.773,0	8	173
Analyte 67	67	191.5	7.82233,0	8	57
Maleimide	68	226.16667	7.88517,0	8	69
Analyte 69	69	0	7.865,0	8	225
Analyte 70	70	0	7.93743,0	7	147
unknown	71	212.2	7.94433,0	8	69
Analyte 72	72	0	7.96417,0	8	163
unknown	73	191	8.01583,0	8	85
2-keto-isovaleric acid 1	74	307.2	8.02778,0	6	89
Analyte 75	75	185.33333	8.04783,0	8	101
Analyte 76	76	0	8.09587,0	5	93
Analyte 77	77	206	8.11033,0	4	157
unknown	78	240	8.11767,0	8	71

Analyte 79	79	399.5	8.16633,0	8	116
Analyte 80	80	0	8.17317,0	8	107
Analyte 81	81	268.5	8.20505,0	7	187
unknown	82	236.71429	8.21083,0	8	57
Analyte 83	83	0	8.22422,0	3	244
Analyte 84	84	213.6	8.29067,0	8	71
Analyte 85	85	0	8.30733,0	4	208
Analyte 86	86	0	8.36417,0	8	116
Analyte 87	87	0	8.36933,0	4	273
unknown	88	200.5	8.39883,0	8	127
Analyte 89	89	7	8.41178,0	3	238
oxalic acid	90	561.75	8.45067,0	8	102
Analyte 91	91	177	8.46133,0	8	299
Analyte 92	92	399.5	8.55683,0	8	147
unknown	93	201.5	8.566,0	7	168
Analyte 94	94	215	8.60486,0	7	130
2-Ketovaleric acid 2	95	373.33333	8.644,0	6	100
unknown	96	267.66667	8.65311,0	3	84
sarcosine	97	687	8.65833,0	4	116
3-Hydroxypropionic acid 1	98	494.5	8.71883,0	8	133
3-Hydroxypyridine	99	717.33333	8.73844,0	3	152
Analyte 100	100	245	8.74638,0	7	267
unknown	101	467	8.75267,0	8	216
unknown	102	443.33333	8.77933,0	3	191
unknown	103	329	8.7804,0	5	191
Analyte 104	104	0	8.80617,0	8	89
Analyte 105	105	196.6	8.84117,0	8	142
Analyte 106	106	167	8.86733,0	4	257
Analyte 107	107	125.66667	8.88033,0	8	57
Analyte 108	108	0	8.90253,0	5	242
unknown	109	379	8.91305,0	7	77
Analyte 110	110	168.71429	8.94183,0	8	174
Analyte 111	111	220	8.95567,0	4	392
3-hydroxybutyric acid	112	675.57143	8.979,0	8	147
unknown	113	456.375	9.01483,0	8	77
unknown	114	406.875	9.047,0	8	281

Analyte 115	115	201	9.05383,0	8	128
sulfuric acid	116	600.875	9.10033,0	8	147
2-amino-2-methylpropane-1,3-diol 2	117	214.75	9.14448,0	7	187
N-Methyl-DL-alanine	118	743.625	9.15167,0	8	130
Analyte 119	119	0	9.188,0	8	207
Analyte 120	120	0	9.22017,0	8	267
Analyte 121	121	0	9.2356,0	5	221
Methyl Phosphate	122	834.875	9.23317,0	8	241
Analyte 123	123	0	9.274,0	7	267
unknown	124	527.25	9.28567,0	4	57
Analyte 125	125	203	9.28981,0	7	282
Analyte 126	126	0	9.38,0	8	211
Analyte 127	127	0	9.38689,0	3	116
Analyte 128	128	188	9.42733,0	4	151
Analyte 129	129	0	9.43044,0	3	171
beta-Alanine 1	130	960	9.4535,0	8	102
Analyte 131	131	0	9.47233,0	8	221
malonic acid 1	132	534	9.51517,0	8	147
Analyte 133	133	175.33333	9.57717,0	8	226
Analyte 134	134	207	9.59457,0	7	57
unknown	135	529.125	9.6065,0	8	147
cycloleucine 2	136	375.5	9.65967,0	8	57
3-Aminoisobutyric acid 2	137	408.5	9.69907,0	5	131
valine	138	974.75	9.7785,0	8	144
Analyte 139	139	433.75	9.825,0	8	103
Methylmalonic acid	140	312.625	9.832,0	8	70
Analyte 141	141	326	9.8515,0	8	281
Analyte 142	142	211	9.87686,0	7	227
Analyte 143	143	162	9.89283,0	8	57
Analyte 144	144	181	9.92667,0	4	156
Analyte 145	145	180	9.91167,0	8	207
unknown	146	335.5	9.9585,0	8	71
Analyte 147	147	334	9.98162,0	7	217
Carnitine	148	313.83333	10.0212,0	8	116
unknown	149	253.5	10.0473,0	7	71
Analyte 150	150	336.25	10.0725,0	8	101
4-hydroxybutyrate	151	894.875	10.078,0	8	147
unknown	152	202.375	10.0988,0	8	71
Analyte 154	154	272.5	10.2131,0	3	151
2-Butyne-1,4-diol	155	281.75	10.2195,0	8	71
2-ketoadipate 3	156	362.5	10.2323,0	8	96

Carbobenzyloxy-L-leucine degr1	157	363.25	10.2555,0	8	100
Dihydroxyacetone	158	660.125	10.2785,0	8	89
unknown	159	197.375	10.301,0	8	57
Analyte 160	160	252	10.3087,0	5	113
benzoic acid	161	787	10.329,0	8	179
unknown	162	172.75	10.3871,0	5	175
unknown	163	176.33333	10.3915,0	8	71
unknown	164	399	10.4464,0	3	143
Analyte 165	165	283.25	10.4738,0	6	318
unknown	166	266.8	10.4713,0	8	163
Ethanolamine	167	826.75	10.4985,0	8	174
Analyte 168	168	199.66667	10.5218,0	8	85
unknown	169	736.14286	10.5591,0	7	50
unknown	170	791.25	10.5673,0	8	158
glycerol	171	682.125	10.6148,0	8	204
unknown	172	347.8	10.6282,0	8	171
phosphate	173	871.25	10.6668,0	8	133
Analyte 174	174	349	10.6749,0	3	51
unknown	175	726.375	10.6668,0	8	158
2-Deoxyuridine	176	280.625	10.714,0	8	71
Analyte 177	177	336.33333	10.7478,0	8	175
unknown	178	227.66667	10.7857,0	5	162
3-hydroxypyruvate	179	345.875	10.8158,0	8	150
unknown	180	251.75	10.8372,0	8	71
4-Vinylphenol	181	220	10.8987,0	4	151
Isoleucine	182	945.625	10.8923,0	8	158
L-Allothreonine 2	183	494.5	10.9153,0	3	341
unknown	184	721.75	10.9767,0	5	161
proline	185	808.75	10.9827,0	4	79
unknown	186	301	10.9753,0	3	221
proline	187	938.375	10.979,0	8	142
unknown	188	750.66667	10.9718,0	3	194
unknown	189	380	11.0192,0	8	71
maleic acid	190	298.14286	11.0342,0	8	370
glycine 2	191	975.75	11.0703,0	8	174
1,4-Cyclohexanedione 2	192	364.25	11.0775,0	8	69

1,3-Cyclohexanedione 2	193	214.4	11.102,0	8	113
unknown	194	243.66667	11.135,0	4	140
unknown	195	333.5	11.1523,0	8	71
succinic acid	196	955.25	11.1802,0	8	147
2,3-Dihydroxypyridine	197	276.375	11.1903,0	8	69
unknown	198	273	11.1976,0	3	281
Bis(2-hydroxypropyl)amine 1	199	292	11.2113,0	3	160
unknown	200	407.71429	11.251,0	7	130
Analyte 201	201	172	11.2732,0	8	99
unknown	202	306.5	11.2847,0	8	315
Analyte 203	203	196	11.2977,0	4	466
unknown	204	234.5	11.3048,0	8	69
Analyte 205	205	219	11.3345,0	8	252
Analyte 206	206	349.25	11.347,0	8	71
Norleucine 1	207	228	11.4048,0	8	215
Thymol	208	315.5	11.4151,0	6	73
Analyte 209	209	133.75	11.4791,0	6	71
Analyte 210	210	0	11.494,0	8	241
Analyte 211	211	0	11.494,0	6	207
Analyte 212	212	154	11.5712,0	8	152
uracil	213	233.625	11.5915,0	8	71
Analyte 214	214	107	11.6173,0	6	80
Citraconic acid 4	215	497.71429	11.6602,0	8	235
fumaric acid	216	949.375	11.6708,0	8	245
unknown	217	142.85714	11.7262,0	8	71
Analyte 218	218	223	11.7533,0	8	340
serine 1	219	961.25	11.7643,0	8	204
Pyrrole-2-Carboxylic Acid	220	316.5	11.775,0	8	241
2,3-Dimethylsuccinic acid	221	476.25	11.782,0	4	293
Pelargonic acid	222	900	11.8343,0	8	117
unknown	223	215	11.8535,0	8	255
unknown	224	309.6	11.8677,0	7	156
Analyte 225	225	170.33333	11.9177,0	8	57
Analyte 226	226	144.5	11.9255,0	8	340
Analyte 227	227	0	11.9557,0	4	152
3-Cyanoalanine	228	739.875	11.953,0	8	141
unknown	229	334.5	11.9611,0	3	242
unknown	230	125.8	12.0176,0	6	57
Analyte 231	231	0	12.0765,0	8	298

threonine 1	232	949.625	12.0977,0	8	219
N-Acetyl-beta-alanine 2	233	240.2	12.2252,0	8	113
Tartronic acid	234	315	12.2413,0	8	191
Analyte 235	235	0	12.2767,0	3	305
Analyte 236	236	226	12.2835,0	7	204
thymine	237	769.875	12.321,0	8	255
Analyte 238	238	53	12.3475,0	8	57
Glutaric Acid	239	355.8	12.3978,0	6	234
Analyte 240	240	306.4	12.4055,0	8	57
Analyte 241	241	139	12.4125,0	8	373
Analyte 242	242	66	12.4232,0	8	191
unknown	243	479	12.4688,0	8	270
DL-Anabasine 1	244	225.5	12.5146,0	7	211
Analyte 245	245	258	12.5269,0	3	229
unknown	246	207.5	12.5432,0	8	57
Analyte 247	247	244.75	12.5522,0	8	99
Analyte 248	248	0	12.5596,0	5	327
Biuret 3	249	258.875	12.5978,0	8	57
Analyte 250	250	242	12.6085,0	8	218
methyl trans-cinnamate	251	205	12.634,0	6	211
aspartic acid 2	252	873.375	12.6533,0	8	160
unknown	253	373.875	12.6663,0	8	258
beta-Alanine 2	254	819.75	12.6795,0	8	248
N-Ethylglycine 1	255	442.5	12.7228,0	8	174
N-Acetyl-L-leucine 3	256	228.33333	12.7777,0	5	218
Maleamate 4	257	402.57143	12.8577,0	8	216
Analyte 258	258	0	12.9611,0	3	218
Erythrose 1	259	529.75	12.9782,0	8	350
unknown	260	246.8	12.9825,0	5	71
5-Methylresorcinol	261	330.375	13.0567,0	8	252
2,4-diaminobutyric acid 3	262	434.42857	13.0616,0	7	128
unknown	263	349.25	13.0823,0	8	174
Capric Acid	264	609.42857	13.0995,0	7	117
Analyte 265	265	209.8	13.1148,0	8	57
Analyte 266	266	0	13.1629,0	6	281
unknown	267	374.25	13.1968,0	8	72
Aminomalonic acid	268	866.625	13.2022,0	8	218
Analyte 269	269	67.33333	13.2664,0	6	239
unknown	270	559.75	13.2875,0	8	68

unknown	271	329.28571	13.296,0	8	73
unknown	272	198.2	13.3471,0	7	71
unknown	273	230.33333	13.361,0	8	160
L-Malic acid	274	941.125	13.4255,0	8	73
unknown	275	304.25	13.4681,0	7	85
Analyte 276	276	351.33333	13.4742,0	7	128
Ethyl cinnamate	277	295.71429	13.485,0	7	159
Analyte 278	278	0	13.5213,0	8	71
Analyte 279	279	407.33333	13.5364,0	5	217
nicotinamide	280	853.375	13.563,0	8	179
unknown	281	426.57143	13.5841,0	7	191
Analyte 282	282	220	13.6347,0	4	57
Threitol	283	914.25	13.642,0	8	217
unknown	284	367.2	13.6892,0	7	102
asparagine 4	285	942.625	13.6962,0	8	100
Analyte 286	286	420.4	13.7322,0	8	186
Analyte 287	287	192.5	13.7717,0	4	84
unknown	288	185.25	13.7693,0	8	71
methionine 1	289	671.75	13.8632,0	8	176
aspartic acid 1	290	922.5	13.8832,0	8	232
unknown	291	746.8	13.9124,0	5	79
trans-4-hydroxy-L-proline 2	292	818.625	13.9405,0	8	304
oxoproline	293	925	13.9617,0	8	58
Analyte 294	294	298.5	13.9589,0	3	211
oxoproline	295	834.42857	13.9647,0	7	254
4-aminobutyric acid 1	296	755	13.9922,0	8	304
Analyte 297	297	191	14.0028,0	7	314
Analyte 298	298	131.8	14.0138,0	8	447
L-glutamic acid	299	915.125	14.0245,0	8	186
unknown	300	345.57143	14.0622,0	8	185
Analyte 301	301	506	14.0717,0	8	292
glutamine 3	302	297.375	14.0935,0	8	71
Analyte 303	303	367.5	14.1138,0	8	245
Analyte 304	304	221	14.1273,0	8	263
2-Amino-2-norbornanecarboxylic acid 1	305	271.2	14.2173,0	6	69

unknown	306	247.16667	14.2137,0	8	85
L-cysteine	307	831	14.2778,0	8	115
Analyte 308	308	375	14.28,0	4	115
Analyte 309	309	0	14.3335,0	8	71
Analyte 310	310	244	14.3362,0	3	111
unknown	311	377.14286	14.3655,0	8	218
Analyte 312	312	274	14.4071,0	7	89
Threonic acid	313	284.25	14.4247,0	4	86
Dodecanol	314	324.5	14.4706,0	7	71
2-hydroxy-3-isopropylbutanedioic acid	315	477	14.4917,0	8	129
alpha-ketoglutaric acid	316	449.83333	14.5153,0	6	192
creatine	317	326.875	14.524,0	8	299
Analyte 318	318	254	14.5304,0	3	82
N(epsilon)-Trimethyllysine	319	230.625	14.5887,0	8	71
glycocyamine 1	320	219.6	14.6063,0	8	273
unknown	321	332.875	14.6192,0	8	142
unknown	322	406.57143	14.6321,0	7	218
Analyte 323	323	155.66667	14.6745,0	5	71
Analyte 324	324	0	14.7835,0	7	235
3-hydroxy-3-methylglutaric acid	325	484.66667	14.8153,0	3	239
threo-beta-hyrdoxyaspartate 2	326	463.28571	14.8248,0	8	188
Tropic Acid	327	360.16667	14.8348,0	7	119
Phenylphosphoric acid	328	478	14.8437,0	8	211
D-erythronolactone 2	329	487.875	14.8603,0	8	231
Digitoxose 2	330	261	14.8752,0	8	113
hexadecane	331	525.375	14.8962,0	8	57
unknown	332	231.42857	14.914,0	7	241
unknown	333	660	14.974,0	8	142
Analyte 334	334	29.83333	15.0296,0	7	244
unknown	335	765.42857	15.0428,0	7	137
glutamic acid	336	828.375	15.0517,0	8	246
Analyte 337	337	0	15.0827,0	8	298
thymidine 2	338	311	15.1403,0	4	299

unknown	339	400.5	15.1498,0	8	218
toluenesulfonic acid	340	439.125	15.175,0	8	229
4-Hydroxybenzoic acid	341	565	15.1849,0	5	223
unknown	342	258.66667	15.1942,0	7	71
Analyte 343	343	288.5	15.2198,0	3	258
unknown	344	404.42857	15.2689,0	7	147
Cytosin	345	406	15.2768,0	8	326
4-hydroxyphenylacetic acid	346	321.71429	15.315,0	8	114
unknown	347	169	15.3479,0	5	380
Fluorene	348	550.66667	15.3504,0	3	380
Analyte 349	349	248.5	15.371,0	8	211
Lyxose 1	350	348.28571	15.4319,0	7	248
lauric acid	351	778.125	15.465,0	8	117
pyrophosphate 3	352	939	15.4795,0	8	451
N-acetyl-L-aspartic acid 1	353	623.125	15.4893,0	8	158
Analyte 354	354	194	15.5428,0	5	128
Analyte 355	355	167	15.5607,0	8	156
1,3-diaminopropane	356	429.125	15.6078,0	8	326
asparagine 1	357	800.875	15.6187,0	8	116
ribose	358	796.125	15.6367,0	8	103
Analyte 359	359	254.57143	15.7088,0	8	57
Ribonic acid, gamma-lactone	360	587.625	15.7365,0	8	115
Analyte 361	361	191.83333	15.7743,0	8	279
Analyte 362	362	132.71429	15.7993,0	8	71
unknown	363	288	15.8573,0	6	188
cyclohexylsulfamic acid 1	364	288	15.8629,0	3	73
Analyte 365	365	300	15.8761,0	7	368
phthalic acid	366	293.5	15.8869,0	3	221
Analyte 367	367	131	15.9167,0	8	282
Analyte 368	368	0	15.9423,0	8	241
xylitol	369	767.5	15.9572,0	8	217
3,6-Anhydro-D-galactose 1	370	250.5	15.9722,0	8	57
unknown	371	278.375	15.9952,0	8	257
N-formyl-L-methionine 2	372	268.25	16.0288,0	8	71
Analyte 373	373	212.6	16.0373,0	6	126
Analyte 374	374	263.5	16.0753,0	4	57

alpha-Aminoadipic acid	375	471	16.1187,0	4	260
Methoxamedrine 2	376	235.8	16.1595,0	8	154
ribitol	377	830.4	16.1673,0	5	94
IS	378	734	16.2017,0	8	226
beta-Glycerophosphoric acid	379	790.66667	16.2038,0	3	299
Acetol 5	380	446.25	16.2147,0	4	79
Analyte 381	381	151	16.2816,0	3	71
unknown	382	221.83333	16.3094,0	7	180
2-Amino-1-phenylethanol	384	651.75	16.3815,0	8	174
unknown	385	295.5	16.4069,0	3	71
Diglycerol 2	386	586	16.4127,0	3	217
Analyte 387	387	212.33333	16.4407,0	8	207
unknown	388	271.66667	16.4813,0	8	248
unknown	389	385.75	16.5173,0	8	71
unknown	390	738.125	16.6043,0	8	96
D-(glycerol 1-phosphate)	391	885.375	16.615,0	8	73
Analyte 392	392	167	16.6487,0	5	266
unknown	393	631.28571	16.6323,0	8	84
Glucose-1-phosphate	394	619.25	16.668,0	8	217
Analyte 395	395	392	16.7361,0	7	207
unknown	397	697.42857	16.7927,0	7	274
2-deoxy-D-glucose 1	398	332.66667	16.8313,0	3	117
O-Phosphorylethanolamine	399	888.375	16.8477,0	8	174
unknown	400	582.25	16.8553,0	4	284
N-Acetyl-L-glutamic acid 2	401	494	16.8948,0	8	84
(2R)-2-amino-3-phosphonopropanoic acid 2	402	245.375	16.93,0	8	156
unknown	403	208.625	16.9635,0	8	71
thymidine 5'-monophosphate degr prod	404	591.71429	16.9727,0	7	81
terephthalic acid	405	361.75	16.9943,0	8	295
unknown	406	283.625	17.016,0	8	69
9-Fluorenone 2	407	389.8	17.0359,0	5	75
unknown	408	304.71429	17.0424,0	7	128
3-phosphoglycerate	409	519.14286	17.0763,0	7	227

hypoxanthine 1	410	927.375	17.178,0	8	265
Cysteinylglycine 3	411	767.5	17.2092,0	8	257
ornithine 1	412	852.625	17.2162,0	8	142
unknown	413	408	17.2873,0	6	174
citric acid	414	371.625	17.2975,0	8	273
citrulline 1	415	637.125	17.3057,0	8	256
alpha-D-glucosamine 1-phosphate	416	669.75	17.3453,0	8	147
Analyte 417	417	359	17.382,0	3	283
O-Phosphoserine 1	418	682	17.3935,0	8	356
Tagatose 1	419	637.875	17.4485,0	8	217
unknown	420	393.75	17.4952,0	8	356
unknown	421	286.375	17.5075,0	8	57
S-carboxymethylcysteine 2	422	303.375	17.5562,0	8	232
Myristic Acid	423	934	17.6035,0	8	117
methionine sulfoxide 1	424	302.5	17.6258,0	8	174
beta-Mannosylglycerate 2	425	516.75	17.6647,0	4	217
unknown	426	216.83333	17.673,0	8	366
O-phosphonothreonine 4	427	251.66667	17.6828,0	5	273
fructose 1	428	912	17.7265,0	8	103
Analyte 429	429	159.125	17.7703,0	8	318
sorbose 1	430	203.28571	17.8155,0	8	149
unknown	431	744.375	17.8247,0	8	103
Gluconic lactone 1	432	636.16667	17.8307,0	6	129
Lumazine	433	353.57143	17.87,0	7	309
Allantoic acid 2	434	420.25	17.9475,0	8	231
unknown	435	319	17.9538,0	8	57
unknown	436	466.71429	17.979,0	7	318
glucose 1	437	866.625	17.9903,0	8	205
tyrosine 2	438	896.875	17.9992,0	8	179
D-Talose 1	439	273.42857	18.0534,0	7	191
dl-p-Hydroxyphenyllactic acid	440	599.6	18.0692,0	5	308
unknown	441	409.5	18.0768,0	8	284
Analyte 442	442	0	18.1144,0	3	55
unknown	443	275.5	18.1317,0	4	235
unknown	444	678.25	18.1512,0	8	87

D-Altrose 1	445	340.25	18.1631,0	5	292
galactose 2	446	828.625	18.1923,0	8	73
unknown	447	230.5	18.2087,0	4	203
unknown	448	322.71429	18.2263,0	8	58
Atrazine-2-hydroxy 5	449	300.875	18.2695,0	8	71
lysine	450	906.625	18.2993,0	8	174
Methyl Palmitoleate	451	446.75	18.312,0	8	81
mannitol	452	710.25	18.326,0	8	205
unknown	453	348	18.374,0	8	86
unknown	454	302.33333	18.3791,0	6	57
sorbitol	455	951.25	18.392,0	8	217
unknown	456	424.125	18.4397,0	8	86
unknown	457	679	18.4455,0	8	159
glucuronic acid 2	458	416.66667	18.4513,0	3	275
tyrosine 1	459	948.25	18.4848,0	8	218
conduritol b epoxide 2	460	685.83333	18.5453,0	6	103
unknown	461	256.28571	18.573,0	8	69
Sedoheptulose	462	351.25	18.578,0	4	159
pentadecanoic acid	463	775.5	18.6028,0	8	117
unknown	464	239.625	18.6328,0	8	85
unknown	465	278.66667	18.662,0	8	72
D-galacturonic acid 2	466	605.625	18.6705,0	8	73
unknown	467	317	18.6963,0	8	85
unknown	468	616	18.7023,0	4	217
1-Hexadecanol	469	764.875	18.725,0	8	75
Analyte 470	470	243	18.7478,0	8	85
3,5-Dihydroxyphenylglycine 2	471	233	18.7627,0	4	283
unknown	472	282	18.7913,0	6	204
Analyte 473	473	0	18.7985,0	8	149
4-hydroxy-3-methoxycinnamaldehyde 2	474	283.5	18.846,0	8	146
Analyte 475	475	216.5	18.8725,0	8	71
N-alpha-Acetyl-L-ornithine 1	476	260	18.92,0	6	415
Analyte 477	477	204	18.9544,0	3	71
Analyte 478	478	339.6	18.9722,0	8	221
pantothenic acid	479	961.5	18.9885,0	8	103

Analyte 480	480	315.5	19.0283,0	8	230
unknown	481	338	19.038,0	6	217
Analyte 482	482	233.5	19.0533,0	6	204
unknown	483	461.75	19.1133,0	8	293
Galactonic acid	484	784.125	19.1542,0	8	73
Guanidinosuccinic acid 3	485	262.16667	19.2222,0	6	297
cytidine-monophosphate 1	486	870.125	19.2528,0	8	243
Analyte 487	487	0	19.2902,0	8	318
unknown	488	792.75	19.3172,0	8	117
unknown	489	289.85714	19.3538,0	7	286
palmitoleic acid	490	910.25	19.3665,0	8	129
Glucosaminic acid	491	245.75	19.408,0	8	294
unknown	492	347.66667	19.5467,0	4	357
unknown	493	306	19.5464,0	3	164
unknown	494	281.33333	19.5653,0	6	369
palmitic acid	495	969.375	19.5802,0	8	117
unknown	496	495.5	19.5903,0	4	220
mucic acid	497	354	19.6263,0	4	174
unknown	498	481.875	19.6903,0	8	205
unknown	499	455.125	19.7088,0	8	73
N-Acetyl-D-galactosamine 1	500	860.5	19.7882,0	8	87
unknown	501	596.375	19.7615,0	8	131
unknown	502	290.14286	19.8113,0	7	326
myo-inositol	503	935.625	19.9148,0	8	318
unknown	504	799.8	19.9177,0	5	58
unknown	505	561.875	19.9832,0	8	87
ribose-5-phosphate 2	506	774	20.0304,0	7	315
unknown	507	698.875	19.9792,0	8	205
unknown	508	258.85714	20.0586,0	7	86
Linoleic acid methyl ester	509	374.125	20.1162,0	8	203
ribulose-5-phosphate 1	510	552.5	20.1337,0	8	357
N-Acetyl-beta-D-mannosamine 4	511	760.125	20.151,0	8	205
unknown	512	365.2	20.1724,0	5	81
unknown	513	320.42857	20.1906,0	7	211
Analyte 514	514	0	20.2223,0	5	132
unknown	515	312.75	20.2666,0	7	129

guanine 1	516	873.375	20.2818,0	8	352
unknown	517	285.8	20.2927,0	8	99
Glucoheptonic acid 3	518	344	20.3157,0	7	211
Analyte 519	519	247.33333	20.3209,0	6	267
cis-Phytol	520	376.33333	20.3513,0	3	87
Analyte 521	521	301.2	20.3563,0	8	86
unknown	522	338.8	20.4563,0	8	103
heptadecanoic acid	523	849.125	20.475,0	8	117
Analyte 524	524	225.33333	20.5056,0	6	85
d-Glucoheptose 2	525	303.5	20.5168,0	8	299
Octadecanol	526	422.625	20.58,0	8	69
Analyte 527	527	283.42857	20.5857,0	8	71
noradrenaline	528	385.5	20.6327,0	5	174
Analyte 529	529	289.75	20.6478,0	8	217
Phytol	530	291.375	20.6767,0	8	85
Indolelactate 1	531	243	20.7307,0	4	73
unknown	532	305.25	20.8033,0	6	424
Analyte 533	533	409	20.8017,0	4	71
unknown	534	506.625	20.83,0	8	103
unknown	535	478.625	20.8693,0	8	283
Analyte 536	536	328	20.9456,0	3	80
Fructose 2,6-biphosphate degr prod 2	537	677.375	21.0205,0	8	73
unknown	538	291	21.046,0	8	203
tryptophan 2	539	319.125	21.088,0	8	314
5-Hydroxyindole-3-acetic acid 1	540	339.75	21.1148,0	8	219
Elaidic acid	541	937.125	21.1358,0	8	117
unknown	542	516.4	21.1407,0	5	211
linoleic acid	544	414	21.1949,0	3	179
oleic acid	545	521	21.2863,0	5	117
unknown	546	444.8	21.2888,0	8	131
Analyte 547	547	340	21.2963,0	8	71
stearic acid	548	968.5	21.3727,0	8	117
pyridoxal phosphate 1	549	322.75	21.4067,0	8	369
unknown	550	264	21.4812,0	7	174
Analyte 551	551	251	21.4961,0	7	57
Analyte 552	552	329.33333	21.5115,0	8	131
indole-3-acetamide 4	553	363.75	21.5603,0	4	221

Analyte 554	554	340	21.6054,0	7	309
Analyte 555	555	350.25	21.7094,0	7	73
fructose-6-phosphate	556	491.5	21.785,0	4	217
unknown	557	383.5	21.82,0	8	125
Analyte 558	558	287	21.8463,0	8	57
unknown	559	471.5	21.9073,0	3	167
unknown	560	305.75	21.9482,0	8	101
Phenyl beta-D-glucopyranoside	561	447.71429	21.9832,0	8	361
unknown	562	359.25	22.0085,0	8	315
unknown	563	464.5	22.039,0	8	217
Analyte 564	564	258.5	22.076,0	6	98
Analyte 565	565	0	22.0938,0	7	186
Analyte 566	566	0	22.1112,0	8	103
Analyte 567	567	0	22.118,0	5	457
glucose-6-phosphate 2	569	530.25	22.1847,0	8	73
unknown	570	356	22.196,0	8	187
Analyte 571	571	0	22.2617,0	8	217
Analyte 572	572	0	22.2909,0	3	85
Analyte 573	573	0	22.294,0	3	85
Analyte 574	574	191	22.4082,0	8	71
arachidonic acid	575	768.5	22.4275,0	8	80
Purine riboside	576	502	22.475,0	8	73
unknown	577	439.2	22.4818,0	8	71
unknown	578	212.66667	22.5167,0	7	258
Analyte 579	579	287	22.5278,0	3	198
unknown	580	367	22.5424,0	7	57
unknown	581	206	22.5522,0	3	126
Analyte 582	582	233.33333	22.5853,0	8	159
Analyte 583	583	0	22.6365,0	8	217
unknown	584	465.5	22.7024,0	3	160
6-phosphogluconic acid	585	721.25	22.7112,0	8	318
N-Acetyl-5-hydroxytryptamine 1	586	437.57143	22.7683,0	7	290
cytidine-5'-monophosphate	587	394.28571	22.7826,0	7	256
Dehydroabietic Acid	588	285.25	22.797,0	4	240
unknown	589	522.375	22.8112,0	8	131
unknown	590	317.5	22.8224,0	3	218
cis-gondoic acid	591	682.125	22.8672,0	8	129
Analyte 592	592	196.5	22.949,0	8	175

uridine 2	593	834.42857	23.0049,0	7	258
Arachidic acid	594	666.75	23.0122,0	8	117
Analyte 595	595	294.75	23.0253,0	8	131
unknown	596	531.125	23.0568,0	8	285
cytidine-monophosphate degr prod	597	357.28571	23.0722,0	8	387
D-erythro-sphingosine 1	598	700.42857	23.1233,0	7	204
unknown	599	506.25	23.1767,0	8	73
saccharopine 3	600	284	23.2182,0	8	358
Analyte 601	601	454	23.2388,0	8	73
unknown	602	314	23.2463,0	8	301
Analyte 603	603	0	23.2629,0	3	250
DL-dihydrosphingosine 1	604	447.5	23.3167,0	8	205
Neohesperidin	605	547.625	23.4368,0	8	217
Analyte 606	606	0	23.4995,0	8	55
Analyte 607	607	284	23.5302,0	8	131
Analyte 608	608	176	23.5529,0	7	227
piceatannol 2	609	424.66667	23.5789,0	3	430
Analyte 610	610	0	23.6264,0	3	204
unknown	611	202.5	23.6935,0	8	181
unknown	612	329.25	23.728,0	8	301
Analyte 613	613	0	23.7605,0	8	387
Dioctyl phthalate	614	940.625	23.7848,0	8	149
Analyte 615	615	219	23.822,0	8	85
Analyte 616	616	288.66667	23.8293,0	8	209
unknown	617	496.75	23.8563,0	4	387
2-Monopalmitin	618	690.125	23.8627,0	8	217
androsterone 1	619	513	23.9353,0	8	91
unknown	620	419.5	24.003,0	4	79
inosine	621	945.5	23.9985,0	8	73
Analyte 622	622	453.5	24.0678,0	3	79
unknown	623	494.66667	24.07,0	6	79
1-Monopalmitin	624	846.25	24.1193,0	8	371
Analyte 625	625	250	24.138,0	8	85
unknown	626	378.83333	24.1978,0	7	370
unknown	627	325.4	24.2052,0	8	159
Analyte 628	628	312	24.238,0	7	259
Analyte 629	629	228.5	24.2622,0	7	85

Analyte 630	630	0	24.3443,0	8	73
Analyte 631	631	330.8	24.3613,0	8	259
sucrose	632	630.375	24.4205,0	8	361
unknown	633	350	24.43,0	8	58
adenosine	634	903.625	24.4718,0	8	236
Behenic acid	635	649.375	24.5438,0	8	117
unknown	636	282.33333	24.6478,0	3	259
xanthosine	637	874	24.7363,0	4	325
lactose 2	638	946.625	24.8043,0	8	204
lactose 2	639	920.25	24.9325,0	8	204
unknown	640	259	25.0562,0	8	172
11-beta-prostaglandin-F-2-alpha 1	641	413.66667	25.1136,0	3	103
Analyte 642	642	227	25.1417,0	4	358
2-Monoolein	643	510.5	25.1713,0	8	103
Monoolein	644	387.57143	25.2849,0	7	129
unknown	645	423.5	25.3087,0	4	204
Lactobionic Acid 1	646	588.625	25.3748,0	8	73
Prostaglandin E2 2	647	319.625	25.3823,0	8	169
guanosine	648	901.25	25.4443,0	8	324
Monostearin	649	844.75	25.5402,0	8	399
2'-deoxyguanosine 1	650	512.4	25.5484,0	5	280
chrysin	651	268	25.6042,0	3	398
Gentiobiose 1	652	482.33333	25.6113,0	6	204
unknown	653	363.66667	25.6229,0	3	257
5'-methylthioadenosine 1	654	769.25	25.6295,0	8	236
kyotorphin 4	655	314.85714	25.6705,0	8	71
unknown	656	305	25.7511,0	6	221
Palatinose	657	414.85714	25.8625,0	8	169
Analyte 658	658	296.71429	25.8862,0	8	144
Lignoceric acid	659	758	25.975,0	8	117
Uridine monophosphate	660	445.85714	26.0775,0	8	352
unknown	661	331.25	26.1013,0	4	124
Digalacturonic acid 2	662	258.33333	26.1313,0	6	169
Analyte 663	663	159.5	26.2531,0	3	243

6-hydroxy caproic acid trimer	664	226.42857	26.3778,0	7	155
4-Androsten-19-ol-3,17-dione 2	665	329.66667	26.4824,0	6	73
5,7-dihydroxy-3-(4-methoxyphenyl)chromen-4-one	666	303	26.4889,0	6	243
unknown	667	369	26.5921,0	7	105
4',5-dihyrroxy-7-methoxyisoflavone	668	317	26.6936,0	3	155
Analyte 669	669	0	26.8108,0	8	204
unknown	670	406.14286	26.8929,0	7	204
inosine 5'-monophosphate	671	720.71429	26.9569,0	7	169
Galactinol 1	672	445	27.0355,0	8	73
unknown	673	237	27.0749,0	6	364
unknown	674	239	27.166,0	3	227
Cerotinic acid	675	438.5	27.2167,0	3	117
4-Androsten-11beta-ol-3,17-dione 2	676	348.8	27.3068,0	8	299
Hesperitin 2	677	370.75	27.3353,0	4	117
Analyte 678	678	293.25	27.3518,0	8	58
Adenosine 5-monophosphate	679	950.875	27.4972,0	8	169
Analyte 680	680	188.5	27.8087,0	3	155
Tetrahydrocorticosterone 1	681	338	27.8931,0	3	204
Cyclic AMP	682	331.4	27.9142,0	8	169
Analyte 683	683	310	28.0912,0	8	119
5-Dihydrocortisone 1	684	293.33333	28.202,0	3	237
5-Dihydrocortisol 2	685	486.33333	28.2955,0	8	207
3,7,12-Trihydroxycoprostane 1	686	610.75	28.421,0	8	368
unknown	687	580	28.4319,0	5	64
Cortexolone 4	688	201	28.4517,0	7	306
unknown	689	430.375	28.5193,0	8	223
unknown	690	368.25	28.6508,0	8	351
cholesterol	691	473.2	28.81,0	5	255
Analyte 692	692	383.5	28.9001,0	7	133
Analyte 693	693	352	29.1313,0	3	259
Aldosterone 2	694	299.4	29.3313,0	6	309
unknown	695	359.75	30.559,0	4	74
Cholestane-3,5,6-triol, (3beta ,5alpha ,6beta)-	696	454.625	30.5523,0	8	57

Analyte 697	697	0	31.903,0	8	311
Analyte 698	698	264	31.914,0	6	283
Analyte 699	699	0	32.8107,0	4	57
Analyte 700	700	0	32.91,0	8	316
Analyte 701	701	0	33.3765,0	8	297

Additional file 2

Peak	MEAN Con	MEAN Tre	P-VALUE	FOLD CHANGE
valine	0.369325497	0.220223264	0.000336889	1.677050328
palmitic acid	1.239281923	1.043496301	0.007490921	1.187624645
stearic acid	0.913042055	0.744723723	0.003644442	1.226014463
succinic acid	0.221966843	0.143498246	0.031936129	1.546826176
Adenosine 5-monophosphate	0.028628158	0.046667612	0.002532634	0.613448108
Isoleucine	0.49326147	0.317411654	0.008124654	1.554011843
inosine	0.742582504	0.367227755	0.008877922	2.022130665
proline	2.081846849	1.65292906	0.011261625	1.259489533
Myristic Acid	0.024563298	0.018152603	0.008377767	1.353155733
hypoxanthine 1	0.304971229	0.164277902	0.009031754	1.856434894
oxoproline	0.646299289	0.77865743	0.004937477	0.830017495
aspartic acid 1	0.986074378	1.287650168	0.002040026	0.765793693
guanosine	0.011824069	0.005406654	0.008312602	2.186947592
4-hydroxybutyrate	0.023066458	0.010145873	0.000207025	2.273481907
xanthosine	0.001043318	3.40964E-06	5.14527E-05	305.9907382
aspartic acid 2	0.014003142	0.023421349	2.47226E-05	0.597879392
guanine 1	0.029680987	0.012426496	0.002821051	2.388524238
1-Monopalmitin	0.001745563	0.001367033	0.018691885	1.276899289
uridine 2	0.002891198	0.001088588	0.008910189	2.655915141
trans-4-hydroxy-L-proline 2	0.008693277	0.010350678	0.024521811	0.839875132
proline	0.011500824	3.40964E-06	0.013185429	3373.032907
unknown	0.510718222	0.388521089	0.002643777	1.314518662
Galactonic acid	0.008026139	0.005221658	0.002650469	1.537086338
lauric acid	0.008967564	0.006131032	0.003370249	1.462651599
thymine	0.002641439	0.000835765	5.95135E-06	3.160504119
xylitol	0.003322532	0.002672077	0.04540297	1.243426972
1-Hexadecanol	0.00531801	0.002905336	0.004169676	1.830428242
Lignoceric acid	0.002590111	0.002052197	0.002508209	1.262116087
N-Methyl-DL-alanine	0.002191283	0.002713891	0.006603959	0.807431891
2-Monopalmitin	0.001731696	0.0009624	0.004664126	1.799351269
conduritol b epoxide 2	0.000238354	0.001597938	0.014603781	0.149163719
O-Phosphoserine 1	0.000900705	0.000739981	0.040464731	1.217200332
Fructose 2,6-biphosphate degr prod 2	1.540548713	1.253744623	0.019802823	1.228757981
3-hydroxybutyric acid	0.001622782	0.000828426	0.000325965	1.958873232
methionine 1	0.114464002	0.089876199	0.020614982	1.273574126
Arachidic acid	0.005833637	0.003821058	0.001523793	1.526707287
Behenic acid	0.001211306	0.000878552	0.001530522	1.378751725
Glucose-1-phosphate	0.004867377	0.01604672	0.038848916	0.303325342
sulfuric acid	0.137668442	0.105932379	0.005251651	1.299587929

4-Hydroxybenzoic acid	0.000526125	9.13539E-05	0.007994658	5.759198427
oxalic acid	1.095132741	0.849325655	0.026662131	1.289414413
unknown	0.003729364	0.004863945	0.000553575	0.766736531
malonic acid 1	0.052570613	0.045506958	0.045174078	1.155221424
unknown	0.005389438	0.004252564	0.000749772	1.267338692
glucose-6-phosphate 2	0.024173675	0.019016678	0.021608173	1.271182854
unknown	0.00876873	0.007734984	0.032587583	1.133645597
unknown	0.001753127	0.000734541	0.03163579	2.38669881
androsterone 1	0.001243052	0.001707537	0.023126575	0.727979385
2'-deoxyguanosine 1	0.000607918	3.67287E-05	0.001977663	16.55158296
unknown	0.005793985	0.004216396	0.00831953	1.374155794
Analyte 301	0.00490426	0.003727196	0.003787964	1.315804126
D-erythronolactone 2	0.002948893	0.001771553	0.023960844	1.664581035
2-hydroxy-3-isopropylbutanedioic acid	0.011428845	0.008050866	0.005387072	1.419579649
unknown	0.031308125	0.021977865	0.003312095	1.424529862
unknown	0.00333456	0.001738475	0.000557817	1.91809465
Cholestane-3,5,6-triol, (3beta ,5alpha ,6beta)-	0.481810421	0.378454678	0.04016568	1.273099393
DL-dihydrosphingosine 1	0.001726977	0.000705689	0.000110798	2.447219323
Uridine monophosphate	0.000787753	0.000469951	0.016401311	1.676245505
Galactinol 1	0.008991143	0.004490344	0.003070276	2.00232816
Analyte 79	1.303814315	0.864123952	0.003572682	1.508827885
unknown	0.182560354	0.126160626	0.025850471	1.447046983
unknown	0.000375335	0.000297591	0.016764277	1.261242033
unknown	0.002828312	0.001418995	0.007196396	1.993180657
unknown	0.016484713	0.011998398	0.001583241	1.373909469
3-hydroxypyruvate	0.008941715	0.005656244	0.006134388	1.580857282
unknown	0.000720625	0.000490468	0.042302773	1.469258699
Analyte 480	0.000985709	0.000620414	0.011041863	1.588792796
2-ketobutyric acid 2	0.010612147	0.008217586	0.016584344	1.291394783
Analyte 240	0.017819426	0.021996672	0.012245191	0.810096483
S-carboxymethylcysteine 2	0.001348382	0.001017267	0.03415285	1.325494775
Analyte 595	0.012591482	0.009361226	0.000577103	1.345067565
Phytol	0.009709696	0.008720129	0.007879113	1.113480847
Analyte 616	0.001284969	0.000527702	6.97979E-05	2.43502954
unknown	0.008330113	0.005287983	0.003211802	1.575291105
saccharopine 3	0.001405165	0.000825443	0.003425803	1.702317551
Analyte 625	0.006616135	0.00571412	0.03792114	1.157857305
unknown	0.003063457	0.002716893	0.010865241	1.127558832
Glucosaminic acid	0.000752194	0.000302321	0.004957223	2.488069123
Analyte 250	0.001531887	0.00095379	0.004759153	1.606105238
uracil	0.014438662	0.013845299	0.006136563	1.04285668

N-Acetyl-L-leucine 3	0.000394189	6.18154E-05	0.012079341	6.376870043
Analyte 524	0.002304071	0.000498981	0.034976682	4.617553638
Analyte 304	0.010182126	0.008959021	0.028691072	1.136522199
unknown	0.002027679	0.00183032	0.015453851	1.107827431
Analyte 592	0.000635322	0.000346482	0.000173439	1.83363748
Analyte 354	0.000331754	9.05752E-05	0.04611047	3.662744791
Analyte 91	0.00029647	0.000255821	0.049516556	1.158893517
Analyte 238	0.000728523	0.000600767	0.010052642	1.212654552
Analyte 1	0.001362711	0.000904119	0.014481464	1.507225957
Analyte 23	0.000656692	0.000505863	0.017388838	1.298161337
Analyte 26	0.168076021	0.105716352	0.045501798	1.589877228
Analyte 27	0.008739285	0.007811395	0.049552315	1.118786638
Analyte 39	0.013341881	0.011207508	0.000240954	1.190441398
Analyte 42	0.015095896	0.009887113	0.000420935	1.526825453
Analyte 64	0.00098803	0.000340764	0.014881838	2.899457682
Analyte 80	0.09006416	0.05140259	0.040392618	1.752132713
Analyte 104	0.001932992	0.002273872	0.001215609	0.850088496
Analyte 131	0.003590948	0.003343744	0.028631645	1.073930231
Analyte 210	0.137067126	0.053781401	0.000266203	2.548597147
Analyte 566	0.004866846	0.002077234	0.002583743	2.342945133
Analyte 571	0.000717172	0.000428397	0.00056751	1.674084496
Analyte 583	0.068168642	0.027880441	0.00189658	2.445034595
Analyte 669	0.000812562	0.001015656	0.013880406	0.800036397
Analyte 697	0.004890187	0.004088506	0.002500251	1.196081735
Analyte 701	0.002304669	0.001923295	0.009872867	1.19829204

Additional file 3

Pathway	Description	# compounds (dem)	# compounds (all)
hsa01100	Metabolic pathways - Homo sapiens (human)	34	129
hsa00230	Purine metabolism - Homo sapiens (human)	8	14
hsa01230	Biosynthesis of amino acids - Homo sapiens (human)	7	21
hsa02010	ABC transporters - Homo sapiens (human)	7	25
hsa01200	Carbon metabolism - Homo sapiens (human)	6	18
hsa05230	Central carbon metabolism in cancer - Homo sapiens (human)	6	15
hsa00970	Aminoacyl-tRNA biosynthesis - Homo sapiens (human)	5	11
hsa01040	Biosynthesis of unsaturated fatty acids - Homo sapiens (human)	5	8
hsa00061	Fatty acid biosynthesis - Homo sapiens (human)	4	7
hsa00240	Pyrimidine metabolism - Homo sapiens (human)	4	12
hsa00260	Glycine, serine and threonine metabolism - Homo sapiens (human)	4	12
hsa00270	Cysteine and methionine metabolism - Homo sapiens (human)	4	8
hsa01210	2-Oxocarboxylic acid metabolism - Homo sapiens (human)	4	12
hsa04974	Protein digestion and absorption - Homo sapiens (human)	4	11
hsa00290	Valine, leucine and isoleucine biosynthesis - Homo sapiens (human)	3	5
hsa00330	Arginine and proline metabolism - Homo sapiens (human)	3	10
hsa00630	Glyoxylate and dicarboxylate metabolism - Homo sapiens (human)	3	9

hsa00650	Butanoate metabolism - Homo sapiens (human)	3	7
hsa00770	Pantothenate and CoA biosynthesis - Homo sapiens (human)	3	6
hsa04978	Mineral absorption - Homo sapiens (human)	3	9
hsa00030	Pentose phosphate pathway - Homo sapiens (human)	2	5
hsa00071	Fatty acid degradation - Homo sapiens (human)	2	3
hsa00250	Alanine, aspartate and glutamate metabolism - Homo sapiens (human)	2	9
hsa00280	Valine, leucine and isoleucine degradation - Homo sapiens (human)	2	3
hsa00310	Lysine degradation - Homo sapiens (human)	2	8
hsa00410	beta-Alanine metabolism - Homo sapiens (human)	2	8
hsa00640	Propanoate metabolism - Homo sapiens (human)	2	6
hsa00760	Nicotinate and nicotinamide metabolism - Homo sapiens (human)	2	7
hsa00920	Sulfur metabolism - Homo sapiens (human)	2	3
hsa04024	cAMP signaling pathway - Homo sapiens (human)	2	7
hsa04922	Glucagon signaling pathway - Homo sapiens (human)	2	8
hsa00020	Citrate cycle (TCA cycle) - Homo sapiens (human)	1	5
hsa00040	Pentose and glucuronate interconversions - Homo sapiens (human)	1	9
hsa00051	Fructose and mannose metabolism - Homo sapiens (human)	1	3
hsa00052	Galactose metabolism - Homo sapiens (human)	1	10
hsa00062	Fatty acid elongation - Homo sapiens (human)	1	1
hsa00072	Synthesis and degradation of ketone bodies - Homo sapiens (human)	1	1
hsa00130	Ubiquinone and other terpenoid-quinone biosynthesis - Homo sapiens (human)	1	1

hsa00140	Steroid hormone biosynthesis - Homo sapiens (human)	1	7
hsa00190	Oxidative phosphorylation - Homo sapiens (human)	1	4
hsa00220	Arginine biosynthesis - Homo sapiens (human)	1	8
hsa00232	Caffeine metabolism - Homo sapiens (human)	1	1
hsa00340	Histidine metabolism - Homo sapiens (human)	1	2
hsa00350	Tyrosine metabolism - Homo sapiens (human)	1	5
hsa00360	Phenylalanine metabolism - Homo sapiens (human)	1	4
hsa00600	Sphingolipid metabolism - Homo sapiens (human)	1	3
hsa00620	Pyruvate metabolism - Homo sapiens (human)	1	3
hsa00790	Folate biosynthesis - Homo sapiens (human)	1	1
hsa01212	Fatty acid metabolism - Homo sapiens (human)	1	1
hsa04071	Sphingolipid signaling pathway - Homo sapiens (human)	1	4
hsa04080	Neuroactive ligand-receptor interaction - Homo sapiens (human)	1	7
hsa04152	AMPK signaling pathway - Homo sapiens (human)	1	2
hsa04727	GABAergic synapse - Homo sapiens (human)	1	5

Additional file 4

Peak	KEGG COMPOUND name	KEGG Link
2-hydroxypyridine	2-Hydroxypyridine	http://www.kegg.jp/dbget-bin/www_bget?cpd:C02502
2-ketobutyric acid 2	2-ketobutyric acid	http://www.kegg.jp/dbget-bin/www_bget?cpd:C00109
lactic acid	lactic acid	http://www.kegg.jp/dbget-bin/www_bget?cpd:C01432
glycolic acid	glycolic acid	http://www.kegg.jp/dbget-bin/www_bget?cpd:C00160
Maleimide	Maleimide	http://www.kegg.jp/dbget-bin/www_bget?cpd:C07272
2-keto-isovaleric acid 1	NA	NA
oxalic acid	oxalic acid	http://www.kegg.jp/dbget-bin/www_bget?cpd:C00209
2-Ketovaleric acid 2	alpha-Ketovaleric acid	http://www.genome.jp/dbget-bin/www_bget?cpd:C06255
sarcosine	sarcosine	http://www.kegg.jp/dbget-bin/www_bget?cpd:C00213
3-Hydroxypropionic acid 1	3-Hydroxypropionic acid	http://www.kegg.jp/dbget-bin/www_bget?cpd:C01013
3-Hydroxypyridine	NA	NA
3-hydroxybutyric acid	(R)-3-Hydroxybutyric acid	NA
sulfuric acid	sulfuric acid	http://www.kegg.jp/dbget-bin/www_bget?cpd:C00059
2-amino-2-methylpropane-1,3-diol 2	2-Amino-2-methyl-1,3-propandiol	http://www.genome.jp/dbget-bin/www_bget?cpd:C11260
N-Methyl-DL-alanine	NA	NA
Methyl Phosphate	NA	NA
beta-Alanine 1	beta-Alanine	http://www.kegg.jp/dbget-bin/www_bget?cpd:C00099
malonic acid 1	malonic acid	http://www.kegg.jp/dbget-bin/www_bget?cpd:C00383
cycloleucine 2	cycloleucine	http://www.kegg.jp/dbget-bin/www_bget?cpd:C03969
3-Aminoisobutyric acid 2	3-Aminoisobutyric acid	http://www.kegg.jp/dbget-bin/www_bget?cpd:C05145
valine	valine	http://www.kegg.jp/dbget-bin/www_bget?cpd:C16436
Methylmalonic acid	Methylmalonic acid	http://www.kegg.jp/dbget-bin/www_bget?cpd:C02170

Carnitine	Carnitine	http://www.kegg.jp/dbget-bin/www_bget?cpd:C00487
4-hydroxybutyrate	4-Hydroxybutyric acid	NA
2-Butyne-1,4-diol	2-Butyne-1,4-diol	http://www.kegg.jp/dbget-bin/www_bget?cpd:C02497
2-ketoadipate 3	NA	NA
Carbobenzyloxy-L-leucine degr1	NA	NA
Dihydroxyacetone	Dihydroxyacetone	http://www.kegg.jp/dbget-bin/www_bget?cpd:C00184
benzoic acid	benzoic acid	http://www.kegg.jp/dbget-bin/www_bget?cpd:C00180
Ethanolamine	Ethanolamine	http://www.kegg.jp/dbget-bin/www_bget?cpd:C00189
glycerol	glycerol	http://www.kegg.jp/dbget-bin/www_bget?cpd:C00116
phosphate	phosphate	http://www.kegg.jp/dbget-bin/www_bget?cpd:C00009
2-Deoxyuridine	2-Deoxyuridine	http://www.kegg.jp/dbget-bin/www_bget?cpd:C00526
3-hydroxypyruvate	3-Hydroxypyruvate	http://www.kegg.jp/dbget-bin/www_bget?cpd:C00168
4-Vinylphenol	4-vinylphenol	http://www.genome.jp/dbget-bin/www_bget?cpd:C05627
Isoleucine	Isoleucine	http://www.kegg.jp/dbget-bin/www_bget?cpd:C16434
L-Allothreonine 2	L-Allothreonine	http://www.kegg.jp/dbget-bin/www_bget?cpd:C05519
proline	proline	http://www.kegg.jp/dbget-bin/www_bget?cpd:C16435
proline	proline	http://www.kegg.jp/dbget-bin/www_bget?cpd:C16435
maleic acid	maleic acid	http://www.genome.jp/dbget-bin/www_bget?cpd:C01384
glycine 2	glycine	http://www.kegg.jp/dbget-bin/www_bget?cpd:C00037
1,4-Cyclohexanedione 2	1,4-Cyclohexanedione	http://www.genome.jp/dbget-bin/www_bget?cpd:C08063
1,3-Cyclohexanedione 2	1,3-Cyclohexanedione	http://www.genome.jp/dbget-bin/www_bget?cpd:C01066
succinic acid	succinic acid	http://www.kegg.jp/dbget-bin/www_bget?cpd:C00042
2,3-Dihydroxypyridine	NA	NA

Bis(2-hydroxypropyl)amine 1	NA	NA
Norleucine 1	L-Norleucine	NA
Thymol	Thymol	http://www.kegg.jp/dbget-bin/www_bget?cpd:C09908
uracil	uracil	http://www.kegg.jp/dbget-bin/www_bget?cpd:C00106
Citraconic acid 4	Citraconic acid	http://www.kegg.jp/dbget-bin/www_bget?cpd:C02226
fumaric acid	fumaric acid	http://www.kegg.jp/dbget-bin/www_bget?cpd:C00122
serine 1	serine	http://www.kegg.jp/dbget-bin/www_bget?cpd:C00065
Pyrrole-2-Carboxylic Acid	Pyrrole-2-Carboxylic Acid	NA
2,3-Dimethylsuccinic acid	NA	NA
Pelargonic acid	Pelargonic acid	http://www.kegg.jp/dbget-bin/www_bget?cpd:C01601
3-Cyanoalanine	L-3-Cyanoalanine	NA
threonine 1	L-Threonine	NA
N-Acetyl-beta-alanine 2	N-Acetyl-beta-alanine	http://www.kegg.jp/dbget-bin/www_bget?cpd:C01073
Tartronic acid	Tartronic acid	http://www.genome.jp/dbget-bin/www_bget?cpd:C02287
thymine	thymine	http://www.kegg.jp/dbget-bin/www_bget?cpd:C00178
Glutaric Acid	glutaric acid	http://www.kegg.jp/dbget-bin/www_bget?cpd:C00489
DL-Anabasine 1	Anabasine	NA
Biuret 3	Biuret	http://www.kegg.jp/dbget-bin/www_bget?cpd:C06555
methyl trans-cinnamate	NA	NA
aspartic acid 2	aspartic acid	http://www.kegg.jp/dbget-bin/www_bget?cpd:C16433
beta-Alanine 2	beta-Alanine	http://www.kegg.jp/dbget-bin/www_bget?cpd:C00099
N-Ethylglycine 1	N-Ethylglycine	http://www.kegg.jp/dbget-bin/www_bget?cpd:C11735
N-Acetyl-L-leucine 3	N-Acetyl-L-leucine	http://www.genome.jp/dbget-bin/www_bget?cpd:C02710
Maleamate 4	Maleamate	http://www.kegg.jp/dbget-bin/www_bget?cpd:C01596

Erythrose 1	D-Erythrose	NA
5-Methylresorcinol	5-Methylresorcinol	http://www.kegg.jp/dbget-bin/www_bget?cpd:C00727
2,4-diaminobutyric acid 3	NA	NA
Capric Acid	n-Capric acid	http://www.genome.jp/dbget-bin/www_bget?cpd:C01571
Aminomalonic acid	Aminomalonic acid	http://www.kegg.jp/dbget-bin/www_bget?cpd:C00872
L-Malic acid	L-Malic acid	http://www.kegg.jp/dbget-bin/www_bget?cpd:C00149
Ethyl cinnamate	Ethyl cinnamate	NA
nicotinamide	nicotinamide	NA
Threitol	NA	NA
asparagine 4	L-Asparagine	http://www.kegg.jp/dbget-bin/www_bget?cpd:C16438
methionine 1	methionine	http://www.kegg.jp/dbget-bin/www_bget?cpd:C00073
aspartic acid 1	aspartic acid	http://www.kegg.jp/dbget-bin/www_bget?cpd:C16433
trans-4-hydroxy-L-proline 2	trans-4-hydroxy-L-proline	http://www.kegg.jp/dbget-bin/www_bget?cpd:C01157
oxoproline	4-Oxoproline	NA
oxoproline	4-Oxoproline	NA
4-aminobutyric acid 1	4-aminobutyric acid	http://www.kegg.jp/dbget-bin/www_bget?cpd:C00334
L-glutamic acid	L-Glutamic acid	http://www.genome.jp/dbget-bin/www_bget?cpd:C00025
glutamine 3	glutamine	http://www.kegg.jp/dbget-bin/www_bget?cpd:C00303
2-Amino-2-norbornanecarboxylic acid 1	NA	NA
L-cysteine	L-cysteine	http://www.kegg.jp/dbget-bin/www_bget?cpd:C00097
Threonic acid	NA	NA
Dodecanol	1-Dodecanol	NA
2-hydroxy-3-isopropylbutanedioic acid	NA	NA
alpha-ketoglutaric acid	alpha-ketoglutaric acid	http://www.kegg.jp/dbget-bin/www_bget?cpd:C00026
creatine	creatine	http://www.kegg.jp/dbget-bin/www_bget?cpd:C00300

N(epsilon)-Trimethyllysine	NA	NA
glycocyamine 1	glycocyamine	http://www.kegg.jp/dbget-bin/www_bget?cpd:C00581
3-hydroxy-3-methylglutaric acid	3-Hydorxy-3-methylglutaric acid	http://www.genome.jp/dbget-bin/www_bget?cpd:C03761
threo-beta-hyrdoxyaspartate 2	NA	NA
Tropic Acid	Tropic Acid	http://www.kegg.jp/dbget-bin/www_bget?cpd:C01456
Phenylphosphoric acid	Phenolic phosphate	http://www.genome.jp/dbget-bin/www_bget?cpd:C02734
D-erythronolactone 2	NA	NA
Digitoxose 2	D-Digitoxose	NA
hexadecane	1,2-Epoxyhexadecane	NA
glutamic acid	D-Glutamic acid	NA
thymidine 2	thymidine	http://www.kegg.jp/dbget-bin/www_bget?cpd:C00214
toluenesulfonic acid	Toluene-4-sulfonate	http://www.genome.jp/dbget-bin/www_bget?cpd:C06677
4-Hydroxybenzoic acid	4-Hydroxybenzoic acid	http://www.kegg.jp/dbget-bin/www_bget?cpd:C00156
Cytosin		
4-hydroxyphenylacetic acid	4-hydroxyphenylacetic acid	http://www.kegg.jp/dbget-bin/www_bget?cpd:C00642
Fluorene	Fluorene	http://www.genome.jp/dbget-bin/www_bget?cpd:C07715
Lyxose 1	D-Lyxose	NA
lauric acid	lauric acid	http://www.kegg.jp/dbget-bin/www_bget?cpd:C02679
pyrophosphate 3	pyrophosphate	http://www.kegg.jp/dbget-bin/www_bget?cpd:C00013
N-acetyl-L-aspartic acid 1	N-Acetyl-L-aspartic acid	http://www.genome.jp/dbget-bin/www_bget?cpd:C01042
1,3-diaminopropane	1,3-diaminopropane	http://www.kegg.jp/dbget-bin/www_bget?cpd:C00986
asparagine 1	L-Asparagine	http://www.kegg.jp/dbget-bin/www_bget?cpd:C16438
ribose	D-Ribose	http://www.kegg.jp/dbget-bin/www_bget?cpd:C08353
Ribonic acid, gamma-lactone	Ribonic acid, gamma-lactone	http://www.genome.jp/dbget-bin/www_bget?cpd:C01685

cyclohexylsulfamic acid 1	cyclohexylsulfamic acid	http://www.kegg.jp/dbget-bin/www_bget?cpd:C02824
phthalic acid	phthalic acid	NA
xylitol	xylitol	http://www.kegg.jp/dbget-bin/www_bget?cpd:C00379
3,6-Anhydro-D-galactose 1	3,6-Anhydro-D-galactose	NA
N-formyl-L-methionine 2	N-formyl-L-methionine	http://www.kegg.jp/dbget-bin/www_bget?cpd:C03145
alpha-Aminoadipic acid	L-alpha-Aminoadipic acid	http://www.genome.jp/dbget-bin/www_bget?cpd:C00956
Methoxamedrine 2	Methoxamine	http://www.kegg.jp/dbget-bin/www_bget?cpd:C07513
ribitol	ribitol	http://www.kegg.jp/dbget-bin/www_bget?cpd:C00474
beta-Glycerophosphoric acid	beta-Glycerophosphoric acid	http://www.genome.jp/dbget-bin/www_bget?cpd:C02979
Acetol 5	Acetol	http://www.kegg.jp/dbget-bin/www_bget?cpd:C05235
2-Amino-1-phenylethanol	2-Amino-1-phenylethanol	http://www.kegg.jp/dbget-bin/www_bget?cpd:C02735
Diglycerol 2	NA	NA
D-(glycerol 1-phosphate)	D-Glycerol 1-phosphate	http://www.kegg.jp/dbget-bin/www_bget?cpd:C00093
Glucose-1-phosphate	NA	NA
2-deoxy-D-glucose 1	2-Deoxy-D-glucose	http://www.genome.jp/dbget-bin/www_bget?cpd:C00586
O-Phosphorylethanolamine	O-Phosphorylethanolamine	http://www.kegg.jp/dbget-bin/www_bget?cpd:C00346
N-Acetyl-L-glutamic acid 2	N-Acetyl-L-glutamic acid	http://www.genome.jp/dbget-bin/www_bget?cpd:C00624
(2R)-2-amino-3-phosphonopropanoic acid 2	NA	NA
thymidine 5'-monophosphate degr prod	dTMP	http://www.kegg.jp/dbget-bin/www_bget?cpd:C00364
terephthalic acid	Terephthalic acid	http://www.kegg.jp/dbget-bin/www_bget?cpd:C06337
9-Fluorenone 2	9-Fluorenone	http://www.kegg.jp/dbget-bin/www_bget?cpd:C06712
3-phosphoglycerate	3-Phosphoglycerate	http://www.genome.jp/dbget-bin/www_bget?cpd:C00197

hypoxanthine 1	hypoxanthine	http://www.kegg.jp/dbget-bin/www_bget?cpd:C00262
Cysteinylglycine 3	L-Cysteinylglycine	NA
ornithine 1	L-Ornithine	NA
citric acid	Citric acid	http://www.kegg.jp/dbget-bin/www_bget?cpd:C00158
citrulline 1	citrulline	http://www.kegg.jp/dbget-bin/www_bget?cpd:C00327
alpha-D-glucosamine 1-phosphate	D-Glucosamine 1-phosphate	http://www.genome.jp/dbget-bin/www_bget?cpd:C06156
O-Phosphoserine 1	O-Phospho-L-serine	http://www.genome.jp/dbget-bin/www_bget?cpd:C01005
Tagatose 1	D-Tagatose	NA
S-carboxymethylcysteine 2	S-Carboxymethyl-L-cysteine	http://www.genome.jp/dbget-bin/www_bget?cpd:C03727
Myristic Acid	Myristic acid	http://www.genome.jp/dbget-bin/www_bget?cpd:C06424
methionine sulfoxide 1	L-Methionine S-oxide	NA
beta-Mannosylglycerate 2	NA	NA
O-phosphonothreonine 4	O-Phospho-L-threonine	NA
fructose 1	fructose	http://www.kegg.jp/dbget-bin/www_bget?cpd:C01496
sorbose 1	sorbose	http://www.kegg.jp/dbget-bin/www_bget?cpd:C01452
Gluconic lactone 1	Gluconic lactone	http://www.kegg.jp/dbget-bin/www_bget?cpd:C00198
Lumazine	Lumazine	http://www.genome.jp/dbget-bin/www_bget?cpd:C03212
Allantoic acid 2	Allantoic acid	http://www.kegg.jp/dbget-bin/www_bget?cpd:C00499
glucose 1	glucose	http://www.kegg.jp/dbget-bin/www_bget?cpd:C00031
tyrosine 2	tyrosine	http://www.kegg.jp/dbget-bin/www_bget?cpd:C00082
D-Talose 1	D-Talose	http://www.kegg.jp/dbget-bin/www_bget?cpd:C06467
dl-p-Hydroxyphenyllactic acid	p-Hydroxyphenyllactate	http://www.genome.jp/dbget-bin/www_bget?cpd:C03672
D-Altrose 1	D-Altrose	http://www.kegg.jp/dbget-bin/www_bget?cpd:C06464

galactose 2	galactose	http://www.kegg.jp/dbget-bin/www_bget?cpd:C01582
Atrazine-2-hydroxy 5	Hydroxyatrazine	http://www.genome.jp/dbget-bin/www_bget?cpd:C06552
lysine	lysine	http://www.kegg.jp/dbget-bin/www_bget?cpd:C16440
Methyl Palmitoleate	NA	NA
mannitol	mannitol	http://www.kegg.jp/dbget-bin/www_bget?cpd:C00392
sorbitol	sorbitol	http://www.kegg.jp/dbget-bin/www_bget?cpd:C00794
glucuronic acid 2	glucuronic acid	http://www.kegg.jp/dbget-bin/www_bget?cpd:C00191
tyrosine 1	tyrosine	http://www.kegg.jp/dbget-bin/www_bget?cpd:C00082
conduritol b epoxide 2	NA	NA
Sedoheptulose	Sedoheptulose	http://www.kegg.jp/dbget-bin/www_bget?cpd:C02076
pentadecanoic acid	pentadecanoic acid	http://www.kegg.jp/dbget-bin/www_bget?cpd:C16537
D-galacturonic acid 2	D-Galacturonic acid	http://www.genome.jp/dbget-bin/www_bget?cpd:C00333
1-Hexadecanol	1-Hexadecanol	http://www.genome.jp/dbget-bin/www_bget?cpd:C00823
3,5-Dihydroxyphenylglycine 2	3,5-Dihydroxy-phenylglycine	http://www.genome.jp/dbget-bin/www_bget?cpd:C12026
4-hydroxy-3-methoxycinnamaldehyde 2	4-Hydroxy-3-methoxycinnamaldehyde	NA
N-alpha-Acetyl-L-ornithine 1	N-Acetylornithine	http://www.kegg.jp/dbget-bin/www_bget?cpd:C00437
pantothenic acid	pantothenic acid	http://www.kegg.jp/dbget-bin/www_bget?cpd:C00864
Galactonic acid	D-Galactonic acid	NA
Guanidinosuccinic acid 3	Guanidinosuccinic acid	http://www.genome.jp/dbget-bin/www_bget?cpd:C03139
cytidine-monophosphate 1	Cytidine-5'-monophosphate	NA
palmitoleic acid	Palmitoleic acid	http://www.genome.jp/dbget-bin/www_bget?cpd:C08362
Glucosaminic acid	D-Glucosaminic acid	http://www.genome.jp/dbget-bin/www_bget?cpd:C03752

palmitic acid	palmitic acid	http://www.kegg.jp/dbget-bin/www_bget?cpd:C00249
mucic acid	mucic acid	http://www.genome.jp/dbget-bin/www_bget?cpd:C00879
N-Acetyl-D-galactosamine 1	N-Acetyl-D-galactosamine	http://www.genome.jp/dbget-bin/www_bget?cpd:C01132
myo-inositol	myo-inositol	http://www.kegg.jp/dbget-bin/www_bget?cpd:C00137
ribose-5-phosphate 2	ribose-5-phosphate	http://www.genome.jp/dbget-bin/www_bget?cpd:C00117
Linoleic acid methyl ester	NA	NA
ribulose-5-phosphate 1	D-Ribulose 5-phosphate	NA
N-Acetyl-beta-D-mannosamine 4	NA	NA
guanine 1	guanine	http://www.kegg.jp/dbget-bin/www_bget?cpd:C00242
Glucoheptonic acid 3	NA	NA
cis-Phytol	NA	NA
heptadecanoic acid	NA	NA
d-Glucoheptose 2	NA	NA
Octadecanol	NA	NA
noradrenaline	Noradrenaline	NA
Phytol	Phytol	http://www.kegg.jp/dbget-bin/www_bget?cpd:C01389
Indolelactate 1	Indolelactate	http://www.kegg.jp/dbget-bin/www_bget?cpd:C02043
Fructose 2,6-biphosphate degr prod 2	D-Fructose 2,6-bisphosphate	http://www.genome.jp/dbget-bin/www_bget?cpd:C00665
tryptophan 2	tryptophan	http://www.kegg.jp/dbget-bin/www_bget?cpd:C00078
5-Hydroxyindole-3-acetic acid 1	5-Hydroxyindoleacetate	http://www.kegg.jp/dbget-bin/www_bget?cpd:C05635
Elaidic acid	Elaidic acid	http://www.kegg.jp/dbget-bin/www_bget?cpd:C01712
linoleic acid	linoleic acid	http://www.kegg.jp/dbget-bin/www_bget?cpd:C01595
oleic acid	oleic acid	http://www.kegg.jp/dbget-bin/www_bget?cpd:C00712
stearic acid	stearic acid	http://www.kegg.jp/dbget-bin/www_bget?cpd:C01530
pyridoxal phosphate 1	Pyridoxal phosphate	http://www.genome.jp/dbget-bin/www_bget?cpd:C00018

indole-3-acetamide 4	indole-3-acetamide	http://www.kegg.jp/dbget-bin/www_bget?cpd:C02693
fructose-6-phosphate	fructose-6-phosphate	NA
Phenyl beta-D-glucopyranoside	Phenyl beta-D-glucopyranoside	http://www.genome.jp/dbget-bin/www_bget?cpd:C11611
glucose-6-phosphate 2	Aldohexose 6-phosphate	http://www.genome.jp/dbget-bin/www_bget?cpd:C03251
arachidonic acid	arachidonic acid	http://www.kegg.jp/dbget-bin/www_bget?cpd:C00219
Purine riboside	Purine riboside	http://www.genome.jp/dbget-bin/www_bget?cpd:C01736
6-phosphogluconic acid	NA	http://www.genome.jp/dbget-bin/www_bget?cpd:C00345
N-Acetyl-5-hydroxytryptamine 1	N-Acetyl-5-hydroxytryptamine	http://www.genome.jp/dbget-bin/www_bget?cpd:C00978
cytidine-5'-monophosphate	cytidine-5'-monophosphate	NA
Dehydroabietic Acid	Dehydroabietic acid	http://www.genome.jp/dbget-bin/www_bget?cpd:C12078
cis-gondoic acid	NA	NA
uridine 2	Uridine	http://www.genome.jp/dbget-bin/www_bget?cpd:C00299
Arachidic acid	Arachidic acid	http://www.genome.jp/dbget-bin/www_bget?cpd:C06425
cytidine-monophosphate degr prod	Cytidine-5'-monophosphate	NA
D-erythro-sphingosine 1	Sphingosine	http://www.genome.jp/dbget-bin/www_bget?cpd:C00319
saccharopine 3	Saccharopine	http://www.genome.jp/dbget-bin/www_bget?cpd:C00449
DL-dihydrosphingosine 1	dihydrosphingosine	NA
Neohesperidin	Neohesperidin	http://www.genome.jp/dbget-bin/www_bget?cpd:C09806
piceatannol 2	Piceatannol	http://www.genome.jp/dbget-bin/www_bget?cpd:C05901
Dioctyl phthalate	Dioctyl phthalate	http://www.kegg.jp/dbget-bin/www_bget?cpd:C03690
2-Monopalmitin	NA	NA
androsterone 1	androsterone	http://www.kegg.jp/dbget-bin/www_bget?cpd:C00523
inosine	inosine	http://www.kegg.jp/dbget-bin/www_bget?cpd:C00294
1-Monopalmitin	NA	NA

sucrose	sucrose	http://www.kegg.jp/dbget-bin/www_bget?cpd:C00089
adenosine	adenosine	http://www.kegg.jp/dbget-bin/www_bget?cpd:C00212
Behenic acid	Behenic acid	http://www.genome.jp/dbget-bin/www_bget?cpd:C08281
xanthosine	Xanthosine	http://www.genome.jp/dbget-bin/www_bget?cpd:C01762
lactose 2	lactose	http://www.kegg.jp/dbget-bin/www_bget?cpd:C00243
lactose 2	lactose	http://www.kegg.jp/dbget-bin/www_bget?cpd:C00243
11-beta-prostaglandin-F-2-alpha 1	11-epi-Prostaglandin F2alpha	http://www.genome.jp/dbget-bin/www_bget?cpd:C05959
2-Monoolein	NA	NA
Monoolein	NA	NA
Lactobionic Acid 1	NA	NA
Prostaglandin E2 2	Prostaglandin E2	http://www.kegg.jp/dbget-bin/www_bget?cpd:C00584
guanosine	guanosine	http://www.kegg.jp/dbget-bin/www_bget?cpd:C00387
Monostearin	NA	NA
2'-deoxyguanosine 1	2'-Deoxyguanosine	http://www.genome.jp/dbget-bin/www_bget?cpd:C00330
chrysin	Chrysin	http://www.genome.jp/dbget-bin/www_bget?cpd:C10028
Gentiobiose 1	Gentiobiose	http://www.genome.jp/dbget-bin/www_bget?cpd:C08240
5'-methylthioadenosine 1	5'-Methylthioadenosine	http://www.genome.jp/dbget-bin/www_bget?cpd:C00170
kyotorphin 4	kyotorphin	http://www.kegg.jp/dbget-bin/www_bget?cpd:C02993
Palatinose	Palatinose	http://www.genome.jp/dbget-bin/www_bget?cpd:C01742
Lignoceric acid	Lignoceric acid	http://www.genome.jp/dbget-bin/www_bget?cpd:C08320
Uridine monophosphate	Uridine monophosphate	http://www.kegg.jp/dbget-bin/www_bget?cpd:C00105
Digalacturonic acid 2	Digalacturonic acid	http://www.kegg.jp/dbget-bin/www_bget?cpd:C02273
6-hydroxy caproic acid trimer	6-Hydroxyhexanoic acid	http://www.genome.jp/dbget-bin/www_bget?cpd:C06103

4-Androsten-19-ol-3,17-dione 2	19-Hydroxyandrost-4-ene-3,17-dione	http://www.genome.jp/dbget-bin/www_bget?cpd:C05284
5,7-dihydroxy-3-(4-methoxyphenyl)chromen-4-one	Biochanin A	http://www.genome.jp/dbget-bin/www_bget?cpd:C00814
4',5-dihyrroxy-7-methoxyisoflavone	Prunetin	http://www.genome.jp/dbget-bin/www_bget?cpd:C10521
inosine 5'-monophosphate	Inosine 5'-monophosphate	http://www.genome.jp/dbget-bin/www_bget?cpd:C00130
Galactinol 1	Galactinol	http://www.kegg.jp/dbget-bin/www_bget?cpd:C01235
Cerotinic acid	NA	NA
4-Androsten-11beta-ol-3,17-dione 2	4-Androsten-11beta-ol-3,17-dione	http://www.kegg.jp/dbget-bin/www_bget?cpd:C05284
Hesperitin 2	NA	NA
Adenosine 5-monophosphate	NA	NA
Tetrahydrocorticosterone 1	Tetrahydrocorticosterone	http://www.genome.jp/dbget-bin/www_bget?cpd:C05476
Cyclic AMP	Cyclic AMP	http://www.genome.jp/dbget-bin/www_bget?cpd:C00575
5-Dihydrocortisone 1	4,5beta-Dihydrocortisone	http://www.genome.jp/dbget-bin/www_bget?cpd:C05469
5-Dihydrocortisol 2	NA	NA
3,7,12-Trihydroxycoprostane 1	3alpha,7alpha,12alpha-Trihydroxycoprostane	http://www.genome.jp/dbget-bin/www_bget?cpd:C05454
Cortexolone 4	NA	NA
cholesterol	Cholesterol	http://www.genome.jp/dbget-bin/www_bget?cpd:C00187
Aldosterone 2	Aldosterone	http://www.genome.jp/dbget-bin/www_bget?cpd:C01780
Cholestane-3,5,6-triol, (3beta ,5alpha ,6beta)-		

Sup Figure 1

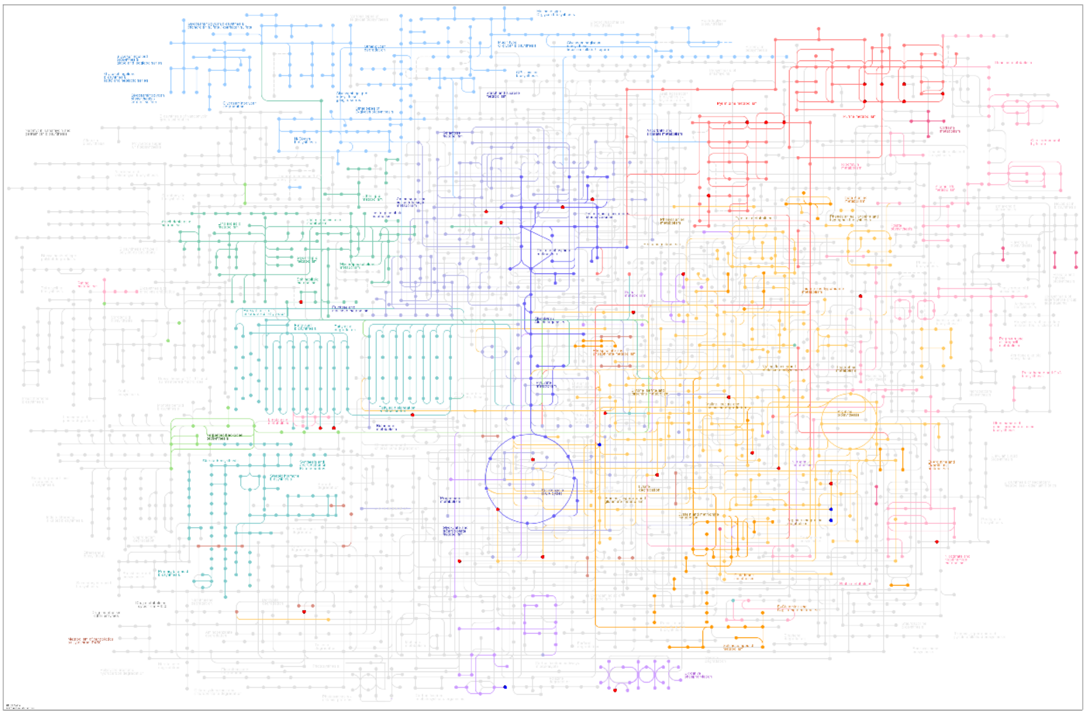

Sup Figure 1: Metabolic pathways with red/blue dots representing the differentially expressed compounds. Bright red dots represented up-regulated metabolites; Bright blue dots represented down-regulated metabolites.
